# *CDK4* is co-amplified with either *TP53* promoter gene fusions or *MDM2* through distinct mechanisms in osteosarcoma

**DOI:** 10.1038/s41525-024-00430-y

**Published:** 2024-09-25

**Authors:** Karim H. Saba, Valeria Difilippo, Emelie Styring, Jenny Nilsson, Linda Magnusson, Hilda van den Bos, René Wardenaar, Diana C. J. Spierings, Floris Foijer, Michaela Nathrath, Felix Haglund de Flon, Daniel Baumhoer, Karolin H. Nord

**Affiliations:** 1 Department of Laboratory Medicine, Division of Clinical Genetics, Lund University, Lund, Sweden; 2 Department of Orthopedics, Lund University, Skåne University Hospital, Lund, Sweden; 3 European Research Institute for the Biology of Ageing, University of Groningen, University Medical Centre Groningen, Groningen, the Netherlands; 4 Children’s Cancer Research Centre and Department of Pediatrics, Klinikum rechts der Isar, Technische Universität München, Munich, Germany; 5 Department of Pediatric Oncology, Klinikum Kassel, Kassel, Germany; 6 Department of Clinical Pathology and Cytology, Karolinska University Hospital, Stockholm, Sweden; 7 Department of Oncology-Pathology, Karolinska Institute, Stockholm, Sweden; 8 Bone Tumour Reference Centre at the Institute of Pathology, University Hospital Basel, Basel, Switzerland; 9 Basel Research Centre for Child Health, Basel, Switzerland

**Keywords:** Bone cancer, Cancer genetics

## Abstract

Amplification of the *MDM2* and *CDK4* genes on chromosome 12 is commonly associated with low-grade osteosarcomas. In this study, we conducted high-resolution genomic and transcriptomic analyses on 33 samples from 25 osteosarcomas, encompassing both high- and low-grade cases with *MDM2* and/or *CDK4* amplification. We discerned four major subgroups, ranging from nearly intact genomes to heavily rearranged ones, each harbouring *CDK4* and *MDM2* amplification or *CDK4* amplification with *TP53* structural alterations. While amplicons involving *MDM2* exhibited signs of an initial chromothripsis event, no evidence of chromothripsis was found in *TP53*-rearranged cases. Instead, the initial disruption of the *TP53* locus led to co-amplification of the *CDK4* locus. Additionally, we observed recurring promoter swapping events involving the regulatory regions of the *FRS2*, *PLEKHA5*, and *TP53* genes. These events resulted in ectopic expression of partner genes, with the *ELF1* gene being upregulated by the *FRS2* and *TP53* promoter regions in two distinct cases.

## Introduction

Osteosarcoma exhibits distinctive subgroups based on clinical, morphological and genetic characteristics. The most prevalent subtype is conventional osteosarcoma, a high-grade tumour necessitating multimodal therapy. Conventional osteosarcoma typically affects children and adolescents but may manifest at any age. The overall survival is up to 70% for patients with localised disease and good response to neoadjuvant chemotherapy, but the prognosis markedly deteriorates for patients with metastatic or recurrent tumours^1–3^. In contrast, low-grade osteosarcoma is rare and generally has a favourable prognosis, with a 5-year survival rate of 90%^4,5^. However, there is a potential for dedifferentiation, leading to the transformation into a high-grade tumour with a prognosis similar to that of conventional osteosarcoma^6,7^. Predominantly affecting adults, with a slight female predominance, low-grade osteosarcoma is further categorised based on tumour site in the bone; low-grade central osteosarcoma originates within the medullary cavity, and parosteal osteosarcoma emerges on the cortical surface^4,5^.

A genetic hallmark of parosteal osteosarcoma is the presence of supernumerary ring or marker chromosomes, often observed as the sole cytogenetically visible alteration^8–10^. These structures encompass amplified material from chromosomal region 12q13-15, housing genes such as *CDK4* and *MDM2*, and occasionally featuring gained or amplified material from chromosome arm 12p as well^8,9,11^. Although studies on low-grade central osteosarcoma are limited, available information suggests the presence of similar amplicons in this subtype, albeit at a lower frequency^12,13^. In contrast, conventional osteosarcoma typically exhibits genome-wide structural and numerical chromosome changes, accompanied by the loss of *TP53* function^14–18^. In approximately 10% of cases, these complex alterations involve the amplification of chromosome bands 12q13-15^11^.

In this study, we conducted comprehensive genomic and transcriptomic analyses on both high- and low-grade osteosarcomas exhibiting *MDM2* and/or *CDK4* amplification and verified our findings using longread whole-genome sequencing. Our aim was to explore whether additional genes within the chromosome 12 amplicons might play a role in tumour progression. Our findings unveiled a correlation between genome-wide alterations and the amplification pattern on chromosome 12. This led to the recognition of four groups, ranging from predominantly intact genomes featuring chromosome 12 amplicons as the sole acquired alteration, to increasingly complex genomes with both *CDK4* and *MDM2* amplification, or *CDK4* co-amplified with *TP53* structural variants. In addition to gene amplification, we observed promoter swapping events that impacted the expression of various known or putative oncogenes.

## Results

**Fig. 1:**
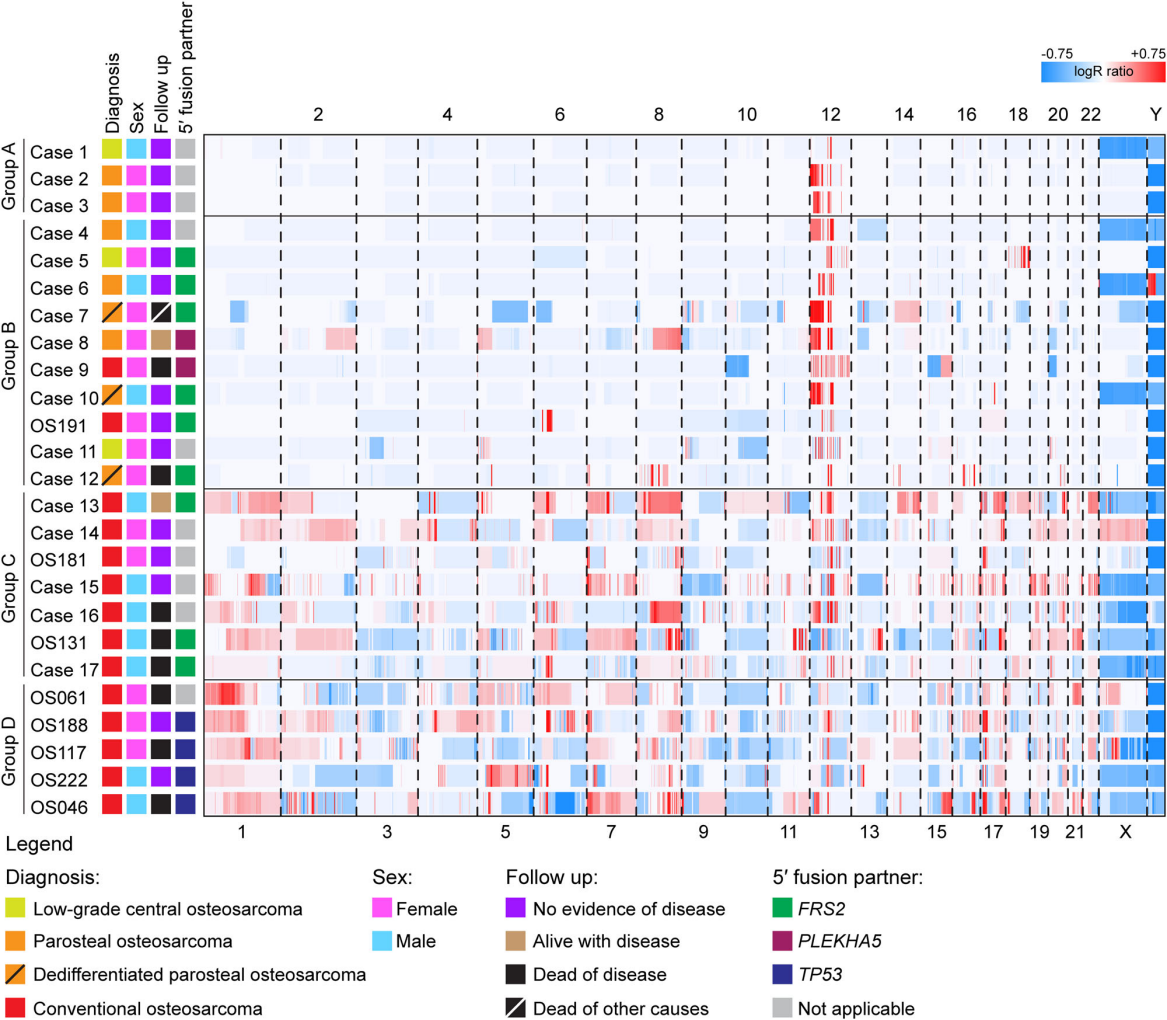
Whole-genome copy number heatmap of 25 osteosarcomas with amplification of 12q sequences. Genome-wide copy number gains (depicted in red) and losses (depicted in blue) were identified by copy number array analysis. The cases are categorised into four groups based on their genome-wide copy number alterations and *MDM2* status. Group A comprises three low-grade osteosarcomas (low-grade central *n* = 1, parosteal *n* = 2) with amplification of *CDK4* and *MDM2* along with other regions of chromosome 12 and no copy number alterations outside of chromosome 12. Group B comprises ten osteosarcomas (including low-grade central *n* = 2, parosteal *n* = 3, dedifferentiated parosteal *n* = 3 and conventional *n* = 2), with amplification of *CDK4* and *MDM2* along with other regions of chromosome 12 and some copy number alterations outside of chromosome 12. Group C comprises seven conventional osteosarcomas with amplification of *CDK4* and *MDM2* and genome-wide copy number alterations. Group D comprises five conventional osteosarcomas with amplification of *CDK4*—but not *MDM2*—and genome-wide copy number alterations.

We selected osteosarcomas based on subtype or *CDK4* and *MDM2* status, drawing cases from two distinct cohorts. Cohort 1 (*n* = 17) comprised a diverse group of osteosarcomas diagnosed at Skåne University Hospital or Karolinska University Hospital in Sweden over the last four decades. Our dataset included DNA copy number and/or RNA sequencing data, revealing amplification and/or relatively high gene expression levels of *CDK4* and/or *MDM2*. Additionally, four cases lacking such information were included due to their diagnosis being parosteal osteosarcoma. Cohort 2 consisted of 108 unselected conventional osteosarcomas for which DNA copy number analyses were available^18^. Among the 108 osteosarcomas, 5% exhibited amplification of *CDK4*, but not *MDM2*, and 3% displayed amplification of both *CDK4* and *MDM2*. In total, we included 33 samples from 25 osteosarcomas in the present study (Supplementary Table 1). The selected cases underwent further exploration using SNP array analysis, bulk tumour whole-genome mate pair and longread sequencing, whole-transcriptome sequencing, and single-cell genome sequencing analyses. Integration of copy number and structural variant data revealed a spectrum of increasing genomic complexity which was then divided into four groups (Fig. 1).

### Group A: *CDK4* and *MDM2* amplified osteosarcomas with no alteration outside of chromosome 12

**Fig. 2:**
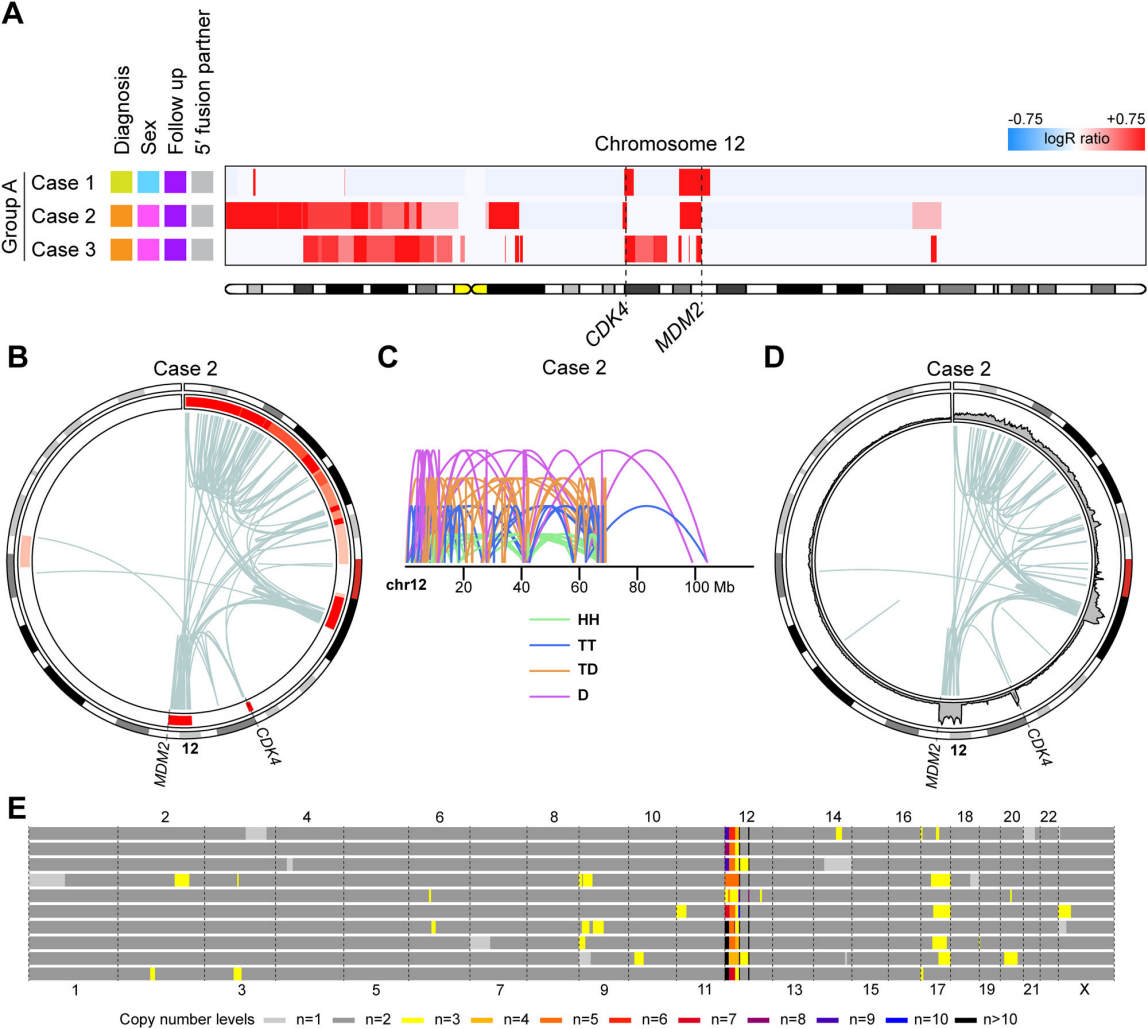
Chromosome 12 copy number heatmap of cases in Group A and combined copy number and structural variant data from a representative case*.* **A** Copy number gains (depicted in red) affecting chromosome 12 were identified by copy number array analysis in three low-grade osteosarcomas. The loci of the *CDK4* and *MDM2* genes are indicated by dashed lines. The colour coding of the sample annotations can be found in Fig. 1. **B** Circos plot of chromosome 12 based on combined copy number and structural variant data. In the circular track under the ideogram, red regions indicate copy number gains and amplifications. Intrachromosomal rearrangements are depicted in light blue. **C** Intrachromosomal structural variants on chromosome 12 are depicted by mapping orientation of the read-pairs. Abbreviations: *HH* head-to-head inversion, *TT* tail-to-tail inversion, *TD* duplication type and *D* deletion type. *Mb* mega-base-pair. **D** Circos plot of chromosome 12 based on longread sequencing data. In the circular track under the ideogram, coverage levels have been plotted as a proxy for copy number levels. Intrachromosomal rearrangements are depicted in light blue. **E** Genome-wide copy numbers of sequenced aberrant cells. Each row represents a single cell.

Group A comprised three low-grade osteosarcomas (one low-grade central and two parosteal) with amplification of *CDK4* and *MDM2*, along with regions on chromosome arm 12p (Figs. 1 and 2A; Supplementary Fig. 1 and 2; Supplementary Table 1). There were no deletions affecting chromosome 12 and no alteration was observed on other chromosomes in bulk DNA. The distribution and read orientation of sequencing reads from chromosome 12 indicated a chromothripsis event (Fig. 2B–D; Supplementary Figs. 1 and 2)^19^. Single-cell whole-genome analysis of one case revealed potential subclones (Fig. 2E; Supplementary Fig. 2). However, the absence of recurrent alterations outside of chromosome 12 suggests that these changes may be attributed to technical artifacts or non-clonal events. In summary, it is likely that Group A amplicons originated from an initial chromothripsis event affecting an extra copy of chromosome 12, followed by subsequent rounds of selective amplification through breakage-fusion-bridge cycles^20–22^.

### Group B: *CDK4* and *MDM2* amplified osteosarcomas with few alterations outside of chromosome 12

**Fig. 3:**
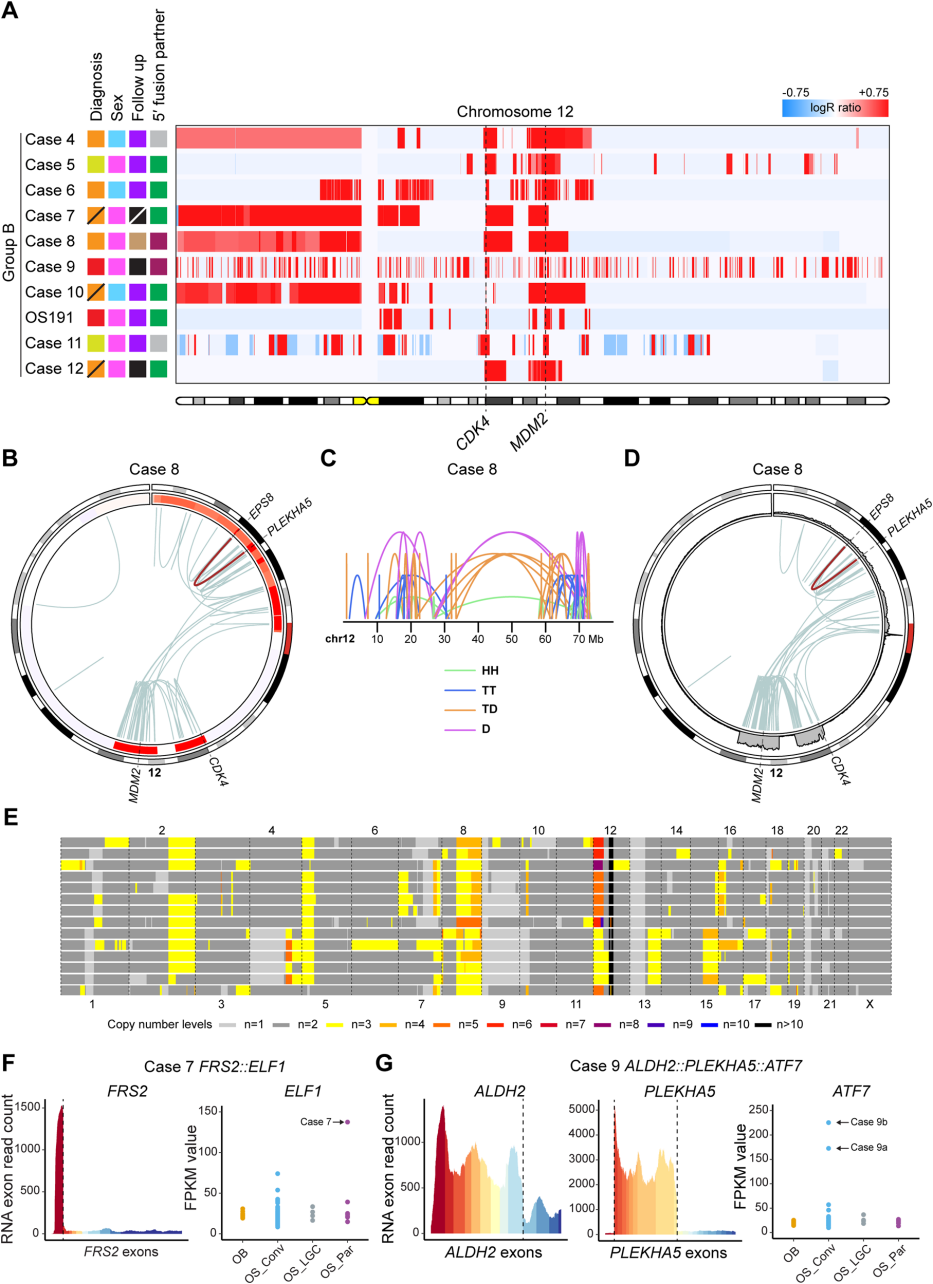
Chromosome 12 copy number heatmap of cases in Group B and combined copy number, structural variant and transcriptomic data from representative cases. **A** Copy number gains (depicted in red) and losses (depicted in blue) affecting chromosome 12 were identified by copy number array analysis in ten low- and high-grade osteosarcomas. The loci of the *CDK4* and *MDM2* genes are indicated by dashed lines. The colour coding of the sample annotations can be found in Fig. 1. **B** Circos plot of chromosome 12 based on combined copy number and structural variant data. In the circular track under the ideogram, red regions indicate copy number gains and amplifications. Intrachromosomal rearrangements are depicted in light blue. A selected variant is depicted in brown. **C** Intrachromosomal structural variants on chromosome 12 are depicted by mapping orientation of the read-pairs. Abbreviations: HH head-to-head inversion, TT tail-to-tail inversion, TD duplication type and D deletion type. Mb mega-base-pair. **D** Circos plot of chromosome 12 based on longread sequencing data. In the circular track under the ideogram, coverage levels have been plotted as a proxy for copy number levels. Intrachromosomal rearrangements are depicted in light blue. A selected variant is depicted in brown. **E** Genome-wide copy numbers of sequenced aberrant cells. Each row represents a single cell. **F** In Case 7, the complete coding sequence of the *ELF1* gene is positioned under the control of the *FRS2* promoter. *FRS2* exon 1 displays a higher expression compared to the exons excluded from the fusion. *ELF1* is upregulated in Case 7 (indicated by an arrow) compared to other osteosarcomas and osteoblastomas. **G** Complex rearrangements in Case 9 lead to the formation of a three-way *ALDH2::PLEKHA5::ATF7* promoter swapping event. The *ALDH2* and *PLEKHA5* exons included in the fusion display a higher expression than the excluded exons. The *ATF7* gene in the multi-sampled Case 9 (indicated by two arrows) is upregulated compared to other osteosarcomas and osteoblastomas. The RNA breakpoints are represented by dashed lines. Abbreviations: *OB* osteoblastoma, *OS_Conv* conventional osteosarcoma, *OS_LGC* low-grade central osteosarcoma and *OS_Par* parosteal or dedifferentiated parosteal osteosarcoma.

Group B included ten osteosarcomas, spanning low- and high-grade cases (three parosteal, two low-grade central, three dedifferentiated parosteal, and two conventional), all displaying amplification of *CDK4* and *MDM2*, along with regions on chromosome arm 12p in all but three cases (Figs. 1 and 3A; Supplementary Figs. 3–13; Supplementary Table 1). There were no deletions affecting chromosome 12, except in one case, and relatively few alterations beyond chromosome 12. The distribution and read orientation of sequencing reads from chromosome 12 suggested a chromothripsis event (Fig. 3B–D; Supplementary Figs. 3–13)^19^. Single-cell whole-genome analyses of three cases indicated the presence of coexisting subclones (Fig. 3E; Supplementary Figs. 4, 8 and 10). Recurrent structural variants affected the *FRS2* (12q15) and *PLEKHA5* (12p12) genes. The 5′ parts of these genes, including their respective promoter region, underwent transposition, leading to elevated expression levels of other genes under their regulatory influence (Fig. 3F–G; Supplementary Figs. 4–11 and 13; Supplementary Tables 2 and 3). In summary, Group B amplicons likely originated from an initial chromothripsis event affecting an extra copy of chromosome 12, followed by subsequent rounds of selective amplification. In some cases, parts of other chromosomes were intertwined with the chromosome 12 amplicons, leading to gene dysregulation through promoter swapping events.

### Group C: *CDK4* and *MDM2* amplified osteosarcomas with extensively altered genomes

**Fig. 4:**
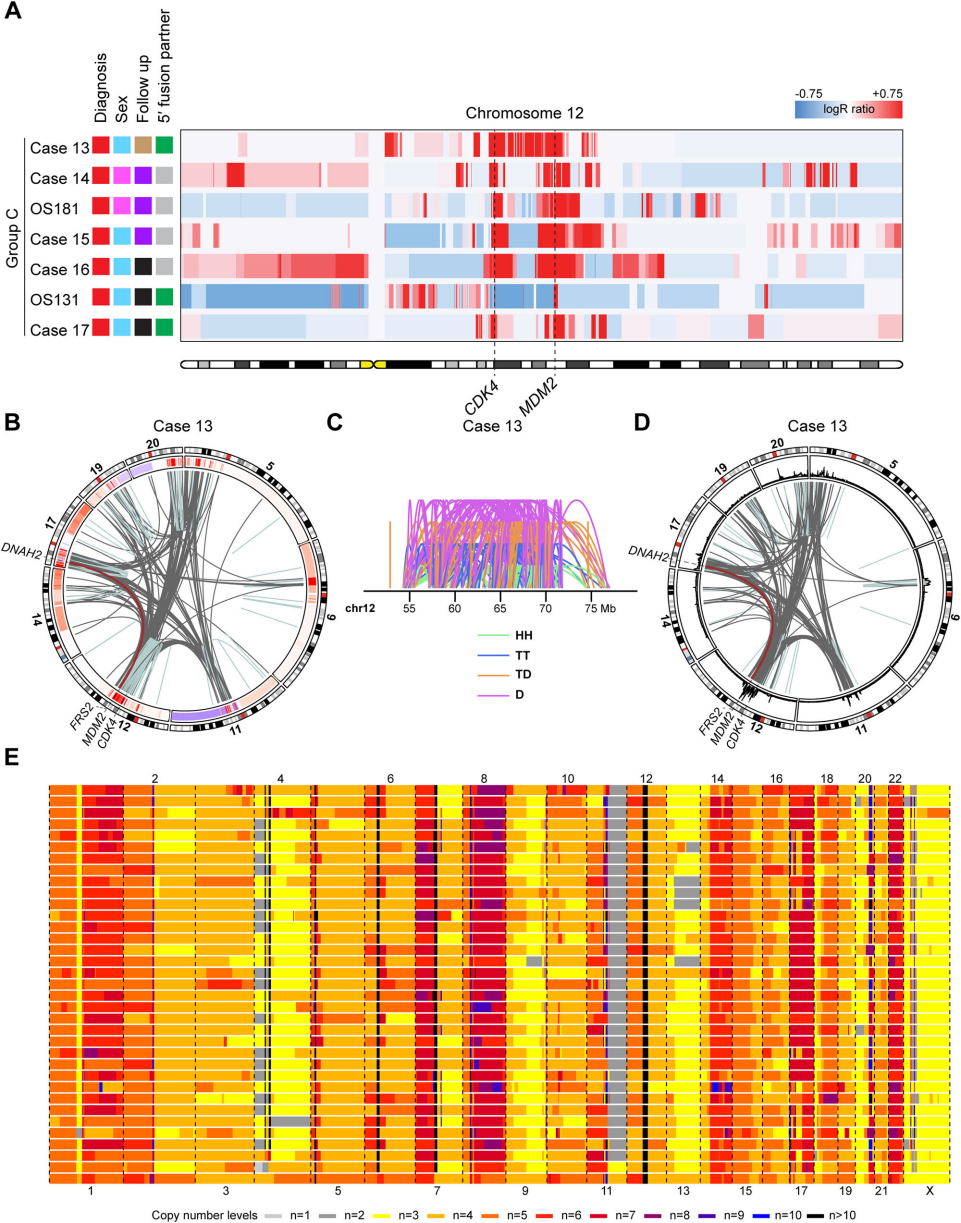
Chromosome 12 copy number heatmap of cases in Group C and combined copy number and structural variant data from a representative case. **A** Copy number gains (depicted in red) and losses (depicted in blue) affecting chromosome 12 were identified by copy number array analysis in seven conventional osteosarcomas. The loci of the *CDK4* and *MDM2* genes are indicated by dashed lines. The colour coding of the sample annotations can be found in Fig. 1. **B** Circos plot of chromosome 12 and selected chromosomes based on combined copy number and structural variant data. In the circular track under the ideograms, red regions indicate copy number gains and amplifications, while blue regions indicate copy number losses. Intrachromosomal and interchromosomal rearrangements are depicted in light blue and grey, respectively. A selected variant is depicted in brown. **C** Intrachromosomal structural variants on chromosome 12 are depicted by mapping orientation of the read-pairs. Abbreviations: *HH* head-to-head inversion, *TT* tail-to-tail inversion, *TD* duplication type and *D* deletion type. *Mb* mega-base-pair. **D** Circos plot of chromosome 12 and selected chromosomes based on longread sequencing data. In the circular track under the ideograms, coverage levels have been plotted as a proxy for copy number levels. Intrachromosomal and interchromosomal rearrangements are depicted in light blue and grey, respectively. A selected variant is depicted in brown. **E** Genome-wide copy numbers of sequenced aberrant cells. Each row represents a single cell.

Group C comprised seven conventional osteosarcomas displaying amplification of *CDK4* and *MDM2*, along with numerous genome-wide copy number and structural variants (Figs. 1 and 4A; Supplementary Figs. 14–19; Supplementary Table 1). Relative copy number loss of parts of chromosome 12 was observed in all but one case. The amplified material from chromosome 12 was intricately intertwined and co-amplified with material from several other chromosomes (Fig. 4B; Supplementary Figs. 14–19). The distribution and read orientation of sequencing reads from chromosome 12 suggested a chromothripsis event in some cases in this group. In the remaining cases, there was inconclusive evidence of chromothripsis affecting chromosome 12, raising the possibility of other mechanisms being responsible for the initial onset of rearrangements and amplifications in these cases (Fig. 4B–D; Supplementary Fig. 18)^19^. Structural variants transposing the *FRS2* promoter region were identified in three cases (Supplementary Table 2). The high genome complexity, coupled with limitations in detecting very high copy numbers, hindered a reliable evaluation of the potential presence of subclones via single-cell sequencing (Fig. 4E; Supplementary Fig. 14 and 19). In summary, it is likely that some Group C amplicons originated from an initial chromothripsis event affecting either an extra copy or one of the normal chromosome 12 homologues, followed by subsequent rounds of selective amplification. Promoter swapping events affecting the *FRS2* gene were detected in both Group B and C.

### Group D: *CDK4*-amplified and *TP53*-rearranged osteosarcomas with extensively altered genomes

**Fig. 5:**
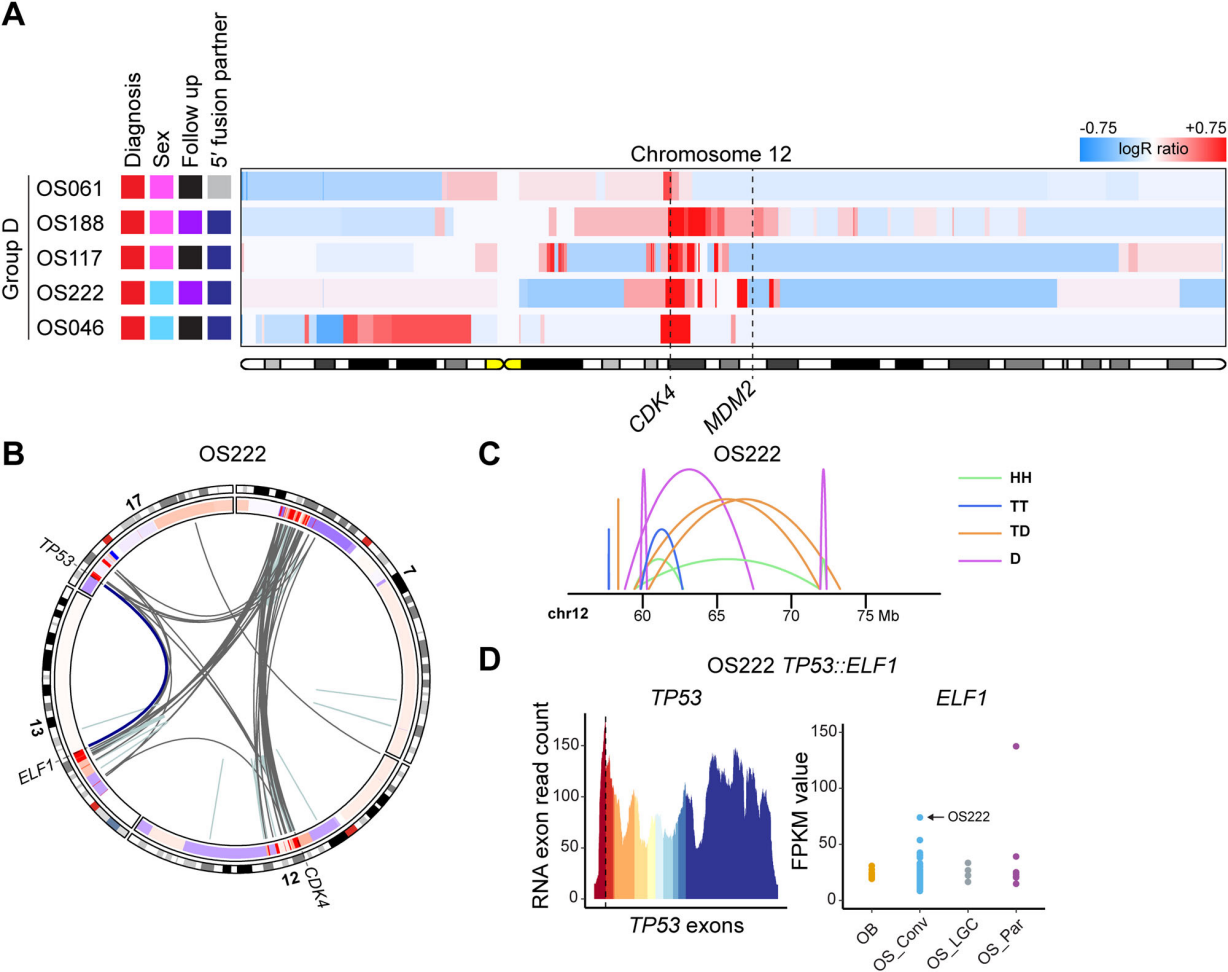
Chromosome 12 copy number heatmap of cases in Group D and combined copy number, structural variant, and transcriptomic data from a representative case. **A** Copy number gains (depicted in red) and losses (depicted in blue) affecting chromosome 12 were identified by copy number array analysis in five conventional osteosarcomas. The loci of the *CDK4* and *MDM2* genes are indicated by dashed lines. The colour coding of the sample annotations can be found in Fig. 1. **B** Circos plot of chromosomes 12, 17 and selected chromosomes based on combined copy number and structural variant data. In the circular track under the ideograms, red regions indicate copy number gains and amplifications, while blue regions indicate copy number losses. Intrachromosomal and interchromosomal rearrangements are depicted in light blue and grey, respectively. A selected variant is depicted in dark blue. **C** Intrachromosomal structural variants on chromosome 12 are depicted by mapping orientation of the read-pairs. Abbreviations: *HH* head-to-head inversion, *TT* tail-to-tail inversion, *TD* duplication type and *D* deletion type. *Mb* mega-base-pair. **D** In OS222, the complete coding sequence of the *ELF1* gene is placed under the control of the *TP53* promoter. *TP53* exon 1 displays a higher expression than the exons excluded from the fusion. The *ELF1* gene in OS222 (indicated by an arrow) is highly expressed compared to other osteosarcomas and osteoblastomas, but lower than in Case 7. The RNA breakpoints are represented by dashed lines. Abbreviations: *OB* osteoblastoma, *OS_Conv* conventional osteosarcoma, *OS_LGC* low-grade central osteosarcoma and *OS_Par* parosteal or dedifferentiated parosteal osteosarcoma.

Group D consisted of five conventional osteosarcomas exhibiting amplification of *CDK4*, rearrangements of *TP53*, and numerous genome-wide copy number and structural variants (Figs. 1 and 5A; Supplementary Figs. 20–22; Supplementary Table 1). In all cases, deletions affecting chromosome 12 were observed. The amplified material from chromosome 12 was intricately intertwined and co-amplified with material from several other chromosomes (Fig. 5B; Supplementary Figs. 20–22). One case harboured a homozygous deletion of *TP53*, while the remaining four cases displayed structural variants transposing the *TP53* promoter region. There was no evidence of a chromothripsis event affecting chromosome 12 in these cases; instead, the initial disruption of the *TP53* locus led to the creation of a dicentric chromosome. Subsequent break and repair processes intertwined and co-amplified the *CDK4* locus with the *TP53* promoter gene fusion, along with parts of other chromosomes involved. In the case with homozygous loss of *TP53*, no clear connection was identified between rearrangements around the lost *TP53* locus and *CDK4* amplification (Fig. 5B, C; Supplementary Fig. 20). The active *TP53* promoter was fused to and upregulated various 3′ partner genes^17^ (Fig. 5D; Supplementary Figs. 21, 22; Supplementary Table 1).

### Osteosarcomas exhibiting *CDK4* amplification comprise of genetic subgroups

All cases presented amplification of *CDK4*. Groups A, B and C exhibited concurrent amplification of *MDM2* and formed a spectrum of increasingly complex genomes, whereas Group D harboured *TP53* promoter gene fusions or *TP53* homozygous loss. For all cases in Groups A and B and most in Group C, an initial chromothripsis event was detected that affected either an extra copy (in case of no deletions affecting chromosome 12) or one of the normal homologues of chromosome 12 (in case of observed deletions affecting chromosome 12). Chromothripsis was detected using a combination of copy number and structural variant data and verified for selected cases using longread whole-genome sequencing where the same patterns of copy number amplification and clustering of structural variants were seen as in the initial analysis (Figs. 2B–D, 3D–B, and 4B–D; Supplementary Figs. 1–19).

**Fig. 6:**
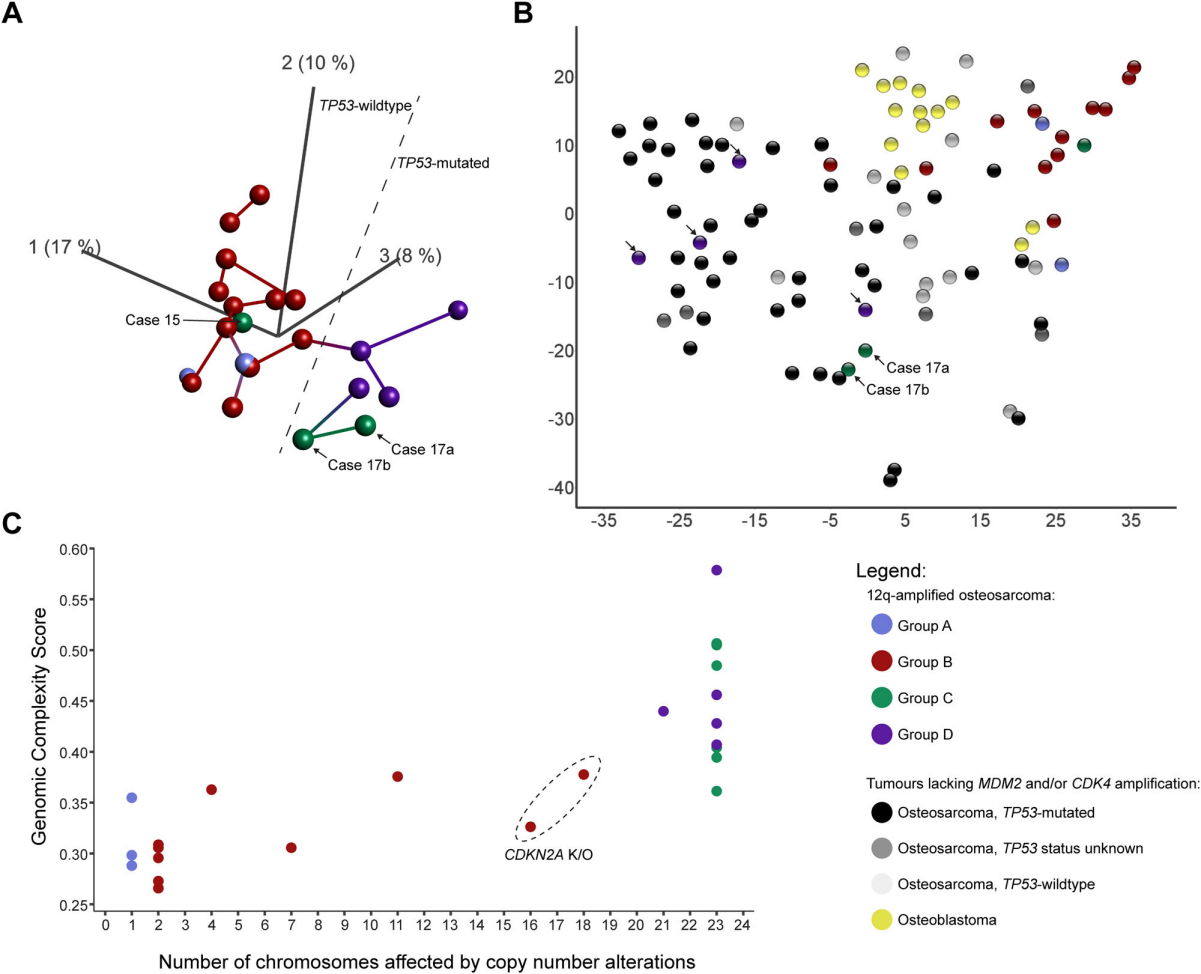
*CDK4*-amplified osteosarcomas show distinct gene expression profiles and diverse genomic complexity levels. **A** Unsupervised principal component analysis based on global gene expression levels in osteosarcomas with amplification of *MDM2* and/or *CDK4* reveal distinct profiles. Cases from Groups B and D are separated in two distinct clusters. However, cases from Group C cluster with both Group B and D. A notable difference in global gene expression is instead determined by the status of the *TP53* gene, where mutated cases cluster distinctly from wildtype cases, assuming all cases have amplification of the *MDM2* and/or *CDK4* genes. The first three principal components representing 17%, 10% and 8% of the variation are displayed. Each sample is connected with its nearest neighbour. Samples to the left of the dashed line are *TP53*-wildtype, while those to the right are *TP53*-mutated. **B** t-distributed stochastic neighbour embedding analysis based on global gene expression levels in all RNA-sequenced osteosarcomas revealed that cases generally separated based on *TP53* mutational status with some exceptions. Osteoblastomas were used as an out-group control. Arrows point to *TP53*-mutated cases with *MDM2* and/or *CDK4* amplification. An unsupervised principal component analysis with the same cases can be seen in Supplementary Movie 1. **C** The genomic complexity score of each case plotted against the number of chromosomes affected by copy number alterations. Cases 7 and 8 from Group B (enclosed within a dashed ellipse), both with complete knock-out of *CDKN2A*, showed copy number alterations on more chromosomes than other cases in Group B. A chromosome was counted as being affected by a copy number alteration if there was a visible copy number shift in the segmentation analysis, or if the chromosome displayed a non-diploid copy number. The X and Y chromosomes were counted as one chromosome pair in males.

Analyses of global gene expression levels revealed that, amongst osteosarcomas with *MDM2* and/or *CDK4* amplification, *TP53*-wildtype tumours clustered separately from *TP53*-mutated tumours (Fig. 6A). This was exemplified in cases from Group C, where *TP53*-wildtype Case 15 clustered with Group B, while Case 17 harbouring two single nucleotide variants in *TP53* clustered with Group D. The remaining samples from Group C lacked RNA-sequencing data, preventing definitive conclusions regarding the effect of *TP53* mutational status on the global expression profile. Upon inclusion of osteoblastomas and osteosarcomas lacking *MDM2* and/or *CDK4* amplification, the tumours formed a spectrum when viewed according to *TP53* mutational status (Fig. 6B; Supplementary Movie 1). Although the *TP53* status could not be determined for all osteosarcomas, *TP53*-mutated cases tended to cluster at one end of the spectrum and *TP53*-wildtype cases at the other end. In individual cases, effects of other mutated genes on the global expression likely also influenced their clustering.

In this dataset, *HMGA2* amplification and *TP53* mutation were observed as mutually exclusive events (Supplementary Table 1; Supplementary Fig. 23). Partial or full amplification of the *HMGA2* gene was found in all cases within Groups A, B and C, except for those in Group C that exhibited concurrent *MDM2* amplification and *TP53* mutation. Notably, *HMGA2* amplification was not detected in Group D.

A genomic complexity score (GCS) was calculated for each case based on DNA copy number data and plotted against the number of chromosomes exhibiting copy number alterations^23^. This analysis revealed two primary clusters among the cases: one characterised by relatively low genetic complexity, with less than half of the chromosomes affected by copy number alterations (except for two cases with homozygous loss of *CDKN2A*); and another with higher genetic complexity, involving nearly all chromosomes displaying copy number alterations (Fig. 6C; Supplementary Table 1). The first cluster included cases assigned to Groups A and B, initially distinguished by whether only chromosome 12 was affected by copy number alterations or if other chromosomes were also affected. The second cluster comprised cases from Groups C and D, exhibiting highly altered genomes and further subdivided based on the presence of either *MDM2* amplification or *TP53* alterations.

In Groups B and C, recurrent rearrangements were observed in the *PLEKHA5* and *FRS2* genes, similar to the *TP53* promoter rearrangements seen in Group D. Specifically, Cases 8 and 9 harboured the *PLEKHA5::EPS8* and *ALDH2::PLEKHA5::ATF7* fusions, respectively, with a 30 kb region of *PLEKHA5* shared between the two fusions at the DNA level (Fig. 3G; Supplementary Fig. 8–10; Supplementary Tables 2 and 3). The functional significance of this shared sequence for the respective *PLEKHA5* fusion remains unclear. Notably, both cases with *PLEKHA5* rearrangements exhibited a particularly aggressive clinical behaviour. However, the low sample size precludes any general conclusions about the aggressiveness of *PLEKHA5*-rearranged tumours at this stage. (Supplementary Table 1). Among the *FRS2*-rearranged cases classified as conventional osteosarcoma, the consequences of a dislocated *FRS2* promoter could not be determined. The remaining five *FRS2*-rearranged cases were low-grade osteosarcomas or of low-grade origin. All *FRS2* promoter partner genes, with one exception, demonstrated the highest expression levels in the affected cases compared with other osteosarcomas (Fig. 3F; Supplementary Figs. 4–7, 11 and 13). In cases with *PLEKHA5* and *FRS2* rearrangements that were multi-sampled, the respective fusions were present in all investigated tumour materials (core needle biopsy, resection specimen, recurrence and/or metastasis), except for Case 12 (*FRS2::ADAM32*). In this case, the fusion was detected in the primary tumour resection but not in the metastatic sample taken almost 20 years later, potentially indicating a slower growth rate without the fusion (Supplementary Tables 1 and 2; Supplementary Fig. 13). It is worth noting that one 3′ fusion partner was recurrent in the present study. The *ELF1* gene was upregulated by both the *FRS2* and *TP53* promoters, resulting in the highest and second highest *ELF1* expression among all 95 RNA-sequenced samples (Figs. 3F and 5D; Supplementary Figs. 7 and 21). However, *ELF1* is not upregulated in other osteosarcomas in this cohort, and its oncogenic role may be limited to rare cases with deregulated expression of the gene.

## Discussion

We demonstrate that genetic rearrangements in osteosarcoma result in ectopic gene expression through two mechanisms: gene amplification and transposition of regulatory elements. Our data support that these events are intertwined and ongoing, at least during tumour initiation. This process facilitates the emergence of subclones with varying fitness, creating a substrate for selection during tumour evolution. In low-grade cases and those with a low-grade origin, this diverse subclonal architecture persists and is detectable at the single cell level – a typical finding previously identified through G-banding analysis^8,24^. Given the potential dedifferentiation of low-grade cases, this emergence of subclones is anticipated. However, in the high-grade cases, such phenomena are more challenging to detect due to the substantial number and magnitude of alterations.

Selective amplification of *CDK4* and *MDM2* is a particularly common phenomenon in low-grade osteosarcoma. However, in high-grade cases, such amplifications are far less frequent^8–11^. Within our unselected cohort of high-grade osteosarcomas, 5% exhibited amplification of *CDK4* alone, and only 3% displayed amplification of both *CDK4* and *MDM2*. It is noteworthy that these amplification patterns are somewhat mirrored in other tumour types. For instance, ring chromosomes containing amplified copies of *MDM2*, often accompanied by *CDK4*, are also present in subtypes of soft tissue tumours. They represent the genetic hallmark of well-differentiated liposarcoma and its dedifferentiated counterpart^9,25–28^. Recently, Sydow et al. suggested that amplification of sequences from chromosome 12 in lipomatous tumours does not occur through classical chromothripsis^19,29^. Instead, they argue that large segments are most often gained after DNA synthesis and that these segments can oscillate between circularised and rod-shaped configurations. When circularised, amplification of selected segments is achieved through repeated breakage-fusion-bridge cycles. Moreover, co-amplification of segments from other chromosomes often appears to occur as a secondary event.

In osteosarcoma, the most biologically significant alteration appears to be the presence of *TP53* mutation, manifested either as structural changes or single nucleotide variants^14–18,30^. In cases with *TP53* structural variants, previous reports indicate that this event occurs early, likely serving as the initiating event in at least half of paediatric osteosarcomas^15,17^. Here, we illustrate that a subset of the *TP53* promoter fusion-positive cases co-amplify *CDK4* during this early process. Unlike *MDM2*-amplified osteosarcomas, *TP53*-rearranged cases show no evidence of chromothripsis affecting chromosome 12. These cases do not seem to benefit from increased copy numbers of *MDM2*, suggesting that altered *TP53* function takes precedence over *MDM2* amplification^17^. Furthermore, the tumour biology differs between *TP53*-rearranged and *MDM2*-amplified osteosarcomas. Generally, *TP53*-rearranged tumours are predominantly found in children and adolescents^15,17^, while *MDM2* amplification is more frequently detected in osteosarcomas of young adults and adults. This difference suggests that *MDM2*-amplified osteosarcoma have distinct origins and biological characteristics compared to *TP53*-rearranged osteosarcomas. For instance, while *MDM2*-amplified osteosarcomas can show concurrent amplification of the *HMGA2* gene, *TP53*-rearranged cases do not^8,27,31–33^. Within the group of *MDM2* and/or *CDK4* amplified osteosarcomas, the *TP53* mutational status seems to have a profound effect on the global gene expression pattern, with *TP53*-mutated osteosarcomas clustering separately from *TP53*-wildtype tumours. When including all 79 transcriptionally analysed osteosarcomas, the trend remained the same, but individual cases demonstrated that factors other than *TP53* status can also have a significant impact on global gene expression. A major obstacle hindering a more comprehensive global gene expression analysis is the rarity of *MDM2*-amplified osteosarcomas and the general lack of publicly available RNA data from osteosarcoma^14–16,30,34–36^.

The backbone of detected amplicons in *MDM2*-amplified osteosarcomas consists mostly of chromosome arm 12q sequences, likely arising from a chromothripsis event affecting chromosome 12. Subsequent rounds of breakage-fusion-bridge cycles lead to copy number amplification^20–22^. In some cases, material from other chromosomes is intertwined and co-amplified through such cycles or possibly through punctuated chromothripsis episodes. Among the co-amplified sequences were the recurrent *FRS2* and *PLEKHA5* gene fusions. Co-amplification of *FRS2* and *MDM2* in low-grade and dedifferentiated osteosarcoma has been previously reported^37^. However, these findings were based on FISH analysis using a probe located 5′ of the *FRS2* gene, making it difficult to assess whether whole or only parts of *FRS2* were amplified. This leaves the possibility open for the amplified copies to be rearranged, leading to *FRS2* fusions similar to what we describe here. We do not have evidence that unequivocally supports a functional role for the *FRS2* and *PLEKHA5* fusion events, and further studies are warranted to determine their role in tumour progression. Supporting a possible biological significance is the fact that these events are recurrent, induce the expression of the 3′ partner genes, and cluster in *MDM2*-amplified osteosarcomas with either aggressive clinical behaviour or dedifferentiated morphology. It should also be noted that the high complexity of these recurrent genetic structures supports, rather than rules out, functional importance, although their function may be more challenging to unravel. An intriguing finding in this context is the fact that the *ELF1* gene was upregulated through fusion to both the *TP53* and the *FRS2* promoters, potentially playing an oncogenic role in rare instances.

In conclusion, our study reveals that *CDK4* is co-amplified with *TP53* promoter gene fusions through mechanisms distinct from those underlying concurrent *CDK4* and *MDM2* amplification. While *TP53* alterations are always accompanied by complex genomic changes, *MDM2*-amplified tumours form a spectrum with varying genetic complexity and additional alterations. These patterns appear to confer biological consequences that manifest as clinically detectable differences, including variations in subtype, age of onset, and outcome. Our findings warrant further investigation of matched genomic and transcriptomic data from osteosarcoma, preferably including rare subtypes such as low-grade central osteosarcoma.

## Methods

### Tumour material and clinical features

The present study encompassed a total of 25 selected cases, with frozen tissue available from more than one time point and/or location for seven of them. The samples comprised diagnostic specimens, primary tumour resections, as well as local and distant recurrences, with the most long-term being obtained 18 years after the initial diagnosis. Additional clinical information on the selected cases is provided in Supplementary Table 1.

### Ethics statement

Informed consent has been obtained from patients and/or their guardians according to the Declaration of Helsinki, and the study was approved by the Swedish Ethics Review Authority (Dnr 2023-01550-01) and the Ethikkommission beider Basel (reference 274/12).

### Genome-wide DNA copy number analyses

DNA was extracted according to standard procedures from fresh frozen tumour biopsies and hybridised to CytoScan HD arrays, following protocols supplied by the manufacturer (Thermo Fisher Scientific, Waltham, MA, USA). Data analysis was performed using the Chromosome Analysis Suite v 4.1.0.90 (Thermo Fisher Scientific), detecting imbalances by visual inspection, and by segmenting log_2_ values using the R packages copynumber^38^ and TAPS^39^. Somatic copy number alterations, based on SNP array data or technologies with lower resolution, have been published previously for a subset of cases (Supplementary Table 1). A GCS was calculated for each case to reflect the degree of genomic complexity based on copy number alterations as previously described^23^.

### Whole-genome mate pair sequencing for detection of structural aberrations

For the detection of structural chromosomal abnormalities, mate pair libraries were prepared for sequencing using the Nextera mate pair sample preparation kit (Illumina, San Diego, CA, USA), as previously described^40^. Sequencing depth was on average 2.72x (mapping coverage 1.96x) and the mean insert size was 3.0 kb, resulting in a median spanning coverage of 53.2x of the human genome (mean 53.0x, range 11.4x-119.1x). All samples were sequenced with high quality and yield; between 13.1 and 115.5 million read pairs were obtained per sample (mean 53.8 million read pairs) and the average quality scores were 31.5-33.5. Sequencing reads were trimmed using NxTrim v 0.4.2^41^ and subsequently aligned against the GRCh37/hg19 build using the Borrows-Wheeler Aligner v 0.7.15^42^. To identify structural rearrangements, the sequence data were analysed using Integrative Genomics Viewer^43,44^, as well as the structural variant callers TIDDIT v 2.12.1, Delly2 v 0.7.8 and Manta v 1.2.2^45–47^. Structural alterations were considered true when identified by at least two of the three variant callers. Copy number and structural variant data were then combined to produce circos plots using the R package circlize^48^ as previously described^17^.

### Whole-genome longread sequencing for verification of structural alterations

For the verification of structural chromosomal abnormalities detected with mate pair whole-genome sequencing, selected samples where high molecular weight DNA could be obtained were subjected to longread whole-genome sequencing on the Pacific Biosciences Revio Technology Platform, using the HiFi protocol (PacBio, Menlo Park, CA, USA). DNA was extracted using the PacBio Nanobind Tissue Kit according to the manufacturer’s instructions. Raw sequencing data was aligned to the GRCh38/hg38 build of the human reference genome using pbmm2 as part of SMRTLink (PacBio) v 13 and structural variants were detected using pbsv. Due to a lack of normal controls, only structural variants annotated as “BND” or “INV” and passing all quality filters were considered for further analyses. Coverage levels were calculated using Mosdepth v 0.3.6^49^ with a bin size of 100,000 bp. Coverage levels and structural variant data were then combined to produce circos plots using the R package circlize^48^ as previously described^17^.

### Whole-genome low-pass sequencing of single cells

Whole-genome sequencing of cryopreserved primary osteosarcoma cells was performed as described in detail previously^50^. In brief, library preparation was performed using a modified single-cell whole-genome sequencing protocol and 77 base pair single reads were generated using a NextSeq 500 sequencing instrument (Illumina). From each assessed case, either 48 or 96 single cells were subjected to sequencing at an average depth of 0.01x and between 29-82 single cells per assessed case passed quality control. This included varying amounts of normal non-neoplastic cells. Copy number analysis was performed using AneuFinder^51^.

### RNA sequencing for detection of gene fusions, expression levels, single nucleotide variants and indels

RNA was extracted according to standard procedures from fresh frozen tumour biopsies and sequenced as previously described^40^. FusionCatcher v 1.0 and STAR-Fusion v 1.4.0 were used to identify candidate fusion transcripts from the sequence data^52,53^. Fusions were considered noise if identified by only one of the programs, or if there was no support for the fusion in whole-genome mate pair sequencing data. NAFuse was used for automatic matching of RNA and DNA sequencing data for gene fusion verification^23^. Sequencing reads were aligned to the GRCh37/hg19 build using STAR v 2.5.2b^54^. For comparison of relative gene expression levels, transcript quantification and normalisation was carried out with RSEM v 1.2.30^55^. Data was visualised using the Qlucore Omics Explorer version 3.8 (Qlucore AB, Lund, Sweden). Relative gene expression levels were evaluated in conventional (*n* = 69), dedifferentiated parosteal (*n* = 3), parosteal (*n* = 4), and low-grade central (*n* = 3) osteosarcomas, as well as osteoblastomas (*n* = 13). For three 12q-amplified samples, RNA-sequencing data from multiple samples was included. Single nucleotide variants and indels were detected using VarScan v 2.4.1, MuTect v 1.1.7 and Mutect2^56–58^. Constitutional variants were excluded based on information from the Genome Aggregation Database (gnomAD v2.1.1)^59^. The detected variants were finally confirmed by manual inspection using the Integrative Genomics Viewer.

### Fluorescence in situ hybridisation

Fluorescence in situ hybridisation (FISH) analyses on metaphase spreads were performed as described previously^60^, using in-house labelled bacterial artificial chromosome clones or commercially available probes specific for the *FRS2* (RP11-1102B16), *CDK4* (RP11-571M6), and *MDM2* (Vysis LSI MDM2) genes, and whole chromosome painting probes (BACPAC Genomics, Emeryville, CA, USA, Applied Spectral Imaging, Carlsbad, CA, USA and Abbott Molecular, Abbott Park, IL, USA).

## Supplementary information

Supplementary Information

Supplementary Movie 1

## Data Availability

Sequencing data for a subset of cases had been previously deposited at the European Genome-Phenome Archive (EGA) under the accession number EGAS00001003842^17^. Data new to this work is deposited at the EGA under the accession numbers EGAS50000000493, EGAS50000000494 and EGAS50000000495.
