# Supplementary material for: CDK4 is co-amplified with either TP53 promoter gene fusions or MDM2 through distinct mechanisms in osteosarcoma
Source: NPJ Genom Med. 2024 Sep 25;9:42. doi: 10.1038/s41525-024-00430-y (PMC11424644; doi:10.1038/s41525-024-00430-y)
Supplement: Supplementary file 1 — Supplementary Information [file 41525_2024_430_MOESM1_ESM.pdf]

Supplementary Information

Supplementary Figure 1. Combined copy number and structural variant data for Case 1.

Supplementary Figure 2. Combined copy number and structural variant data for Case 2.

Supplementary Figure 3. Combined copy number and structural variant data for Case 4.

Supplementary Figure 4. Combined copy number, structural variant and transcriptomic data for Case 5a.

Supplementary Figure 5. Combined copy number, structural variant and transcriptomic data for Case 5b.

Supplementary Figure 6. Combined copy number, structural variant and transcriptomic data for Case 6.

Supplementary Figure 7. Combined copy number, structural variant and transcriptomic data for Case 7.

Supplementary Figure 8. Combined copy number, structural variant and transcriptomic data for Case 8.

Supplementary Figure 9. Transcriptomic data for Case 9a.

Supplementary Figure 10. Combined copy number, structural variant and transcriptomic data for Case 9b.

Supplementary Figure 11. Combined copy number, structural variant and transcriptomic data for Case 10.

Supplementary Figure 12. Combined copy number and structural variant data for OS191.

Supplementary Figure 13. Combined copy number, structural variant and transcriptomic data for Case 12.

Supplementary Figure 14. Combined copy number and structural variant data for Case 13.

Supplementary Figure 15. Combined copy number and structural variant data for Case 14.

Supplementary Figure 16. Combined copy number and structural variant data for Case 15.

Supplementary Figure 17. Combined copy number and structural variant data for Case 16.

Supplementary Figure 18. Combined copy number and structural variant data for OS131.

Supplementary Figure 19. Combined copy number and structural variant data for Case 17.

Supplementary Figure 20. Combined copy number and structural variant data for OS161.

Supplementary Figure 21. Combined copy number, structural variant and transcriptomic data for OS222.

Supplementary Figure 22. Combined copy number, structural variant and transcriptomic data for OS046.

Supplementary Figure 23. *HMGA2* exon coverage plots in selected cases.

Supplementary Figure 24. Histological re-evaluation of Case 1.

Supplementary Table 1. Clinical and genetic features of *MDM2* and/or *CDK4* amplified osteosarcomas.

Supplementary Table 2. Breakpoints affecting the *FRS2* and *PLEKHA5* genes identified by whole genome mate pair and whole-genome longread sequencing.

Supplementary Table 3. *FRS2* and *PLEKHA5* fusion transcripts detected by FusionCatcher and STAR-Fusion.

Supplementary Movie 1. Unsupervised principal component analysis of 79 osteosarcomas and 13 osteoblastomas. The first three principal components representing 10%, 5% and 4% of the variation are displayed. Group A cases are depicted in blue, Group B cases in red, Group C cases in green, and Group D cases in purple. Osteosarcomas lacking *MDM2* and *CDK4* amplification are further divided into *TP53*-mutated cases depicted in black, *TP53*-wildtype cases in light grey, and undetermined *TP53* status in dark grey. Osteoblastomas are depicted in yellow. Arrows point to *TP53*-mutated cases with concurrent *MDM2* and/or *CDK4* amplification. In the PDF version of this article, please click anywhere on the Fig. or caption to play the video in a separate window.

Supplementary Figure 1

Case 1 - Low-grade central osteosarcoma

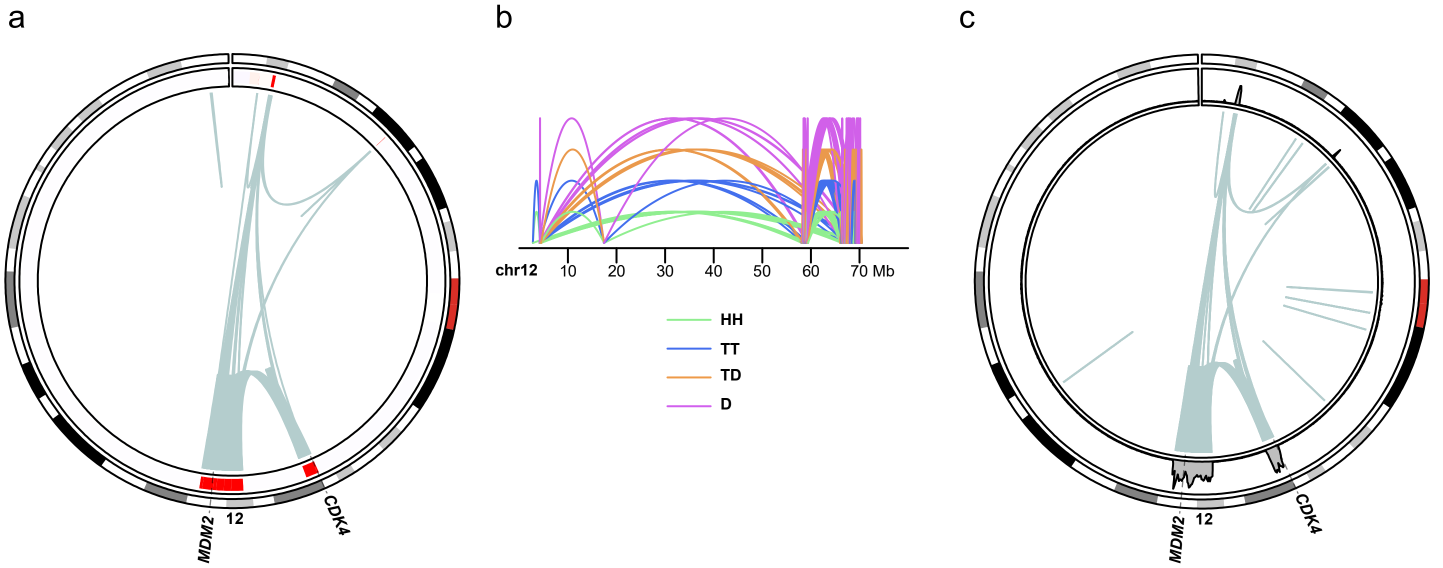

Supplementary Figure 1. Combined copy number and structural variant data for Case 1. a) WGS circos plot: Red regions in the inner circular track of the circos plot indicate copy number gains, with higher level amplifications being in a more intense shade and lower-level gains in a lighter shade. Intrachromosomal structural variants are depicted in light blue. Data is based on copy number array and mate pair whole-genome sequencing. **b) Structural variant distribution:** Intrachromosomal structural variants plotted based on read mapping orientation. Abbreviations: HH = head-to-head inversion, TT = tail-to-tail inversion, TD = duplication type and D = deletion type. Mb = mega-base-pair. **c) Longread WGS circos plot:** Coverage levels are plotted in the inner circular track of the circos plot as a proxy for copy number levels. Intrachromosomal structural variants are depicted in light blue. Data is based on longread whole-genome sequencing. cell.

Supplementary Figure 2

Case 2 - Parosteal osteosarcoma

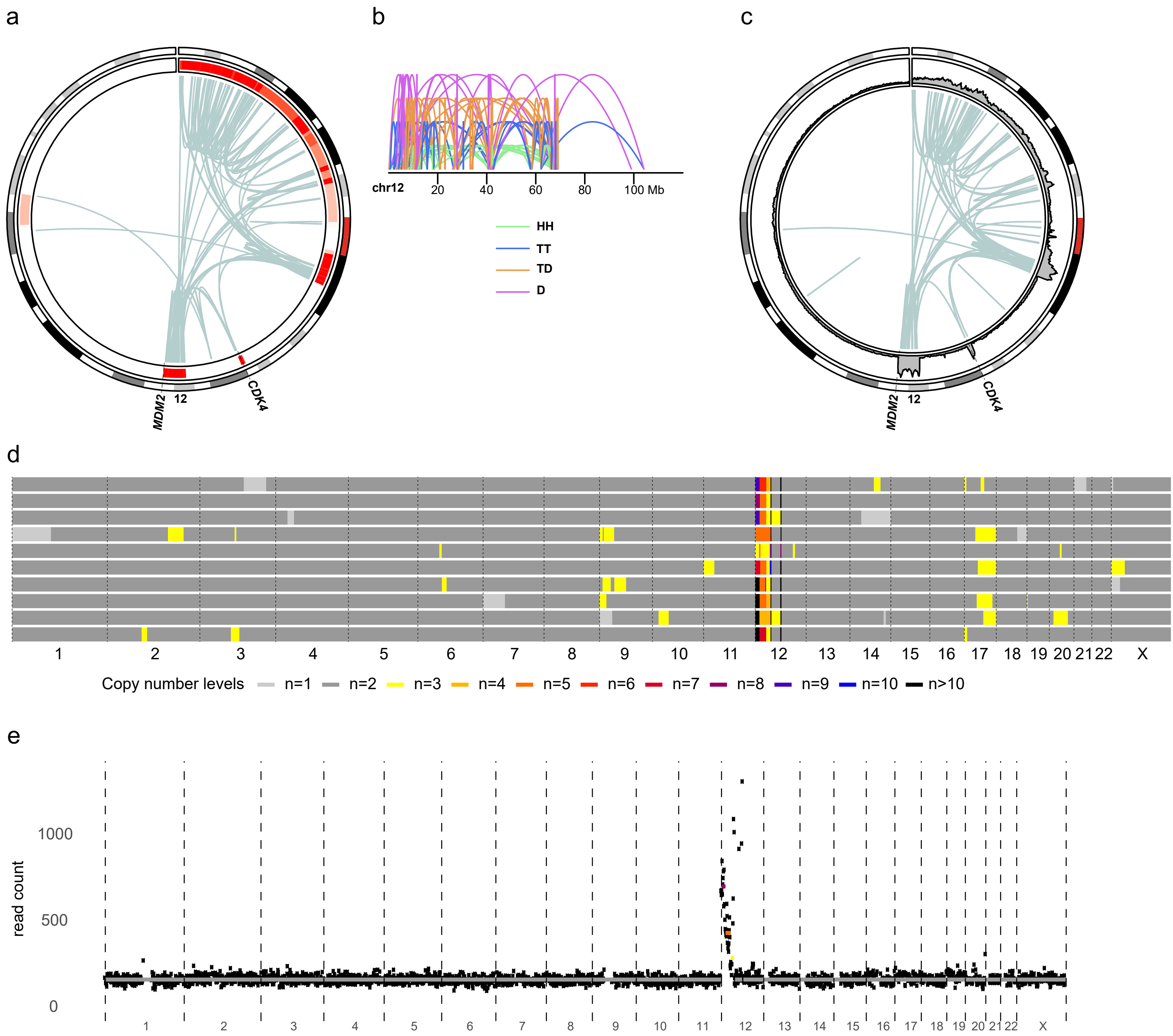

Supplementary Figure 2. Combined copy number and structural variant data for Case 2. a) WGS circos plot: Red regions in the inner circular track of the circos plot indicate copy number gains, with higher level amplifications being in a more intense shade and lower-level gains in a lighter shade. Intrachromosomal structural variants are depicted in light blue. Data is based on copy number array and mate pair whole-genome sequencing. **b) Structural variant distribution:** Intrachromosomal structural variants plotted based on read mapping orientation. Abbreviations: HH = head-to-head inversion, TT = tail-to-tail inversion, TD = duplication type and D = deletion type. Mb = mega-base-pair. **c) Longread WGS circos plot:** Coverage levels are plotted in the inner circular track of the circos plot as a proxy for copy number levels. Intrachromosomal structural variants are depicted in light blue. Data is based on longread whole-genome sequencing. **d) Single cell whole-genome heatmap:** Genome-wide copy numbers of sequenced aberrant cells. Each row represents a single cell. A total of 96 individual cells were sequenced, and non-neoplastic cells were excluded from the heatmap. **e) Representative single cell:** An example whole-genome copy number view of a single cell.

Supplementary Figure 3

Case 4 - Parosteal osteosarcoma

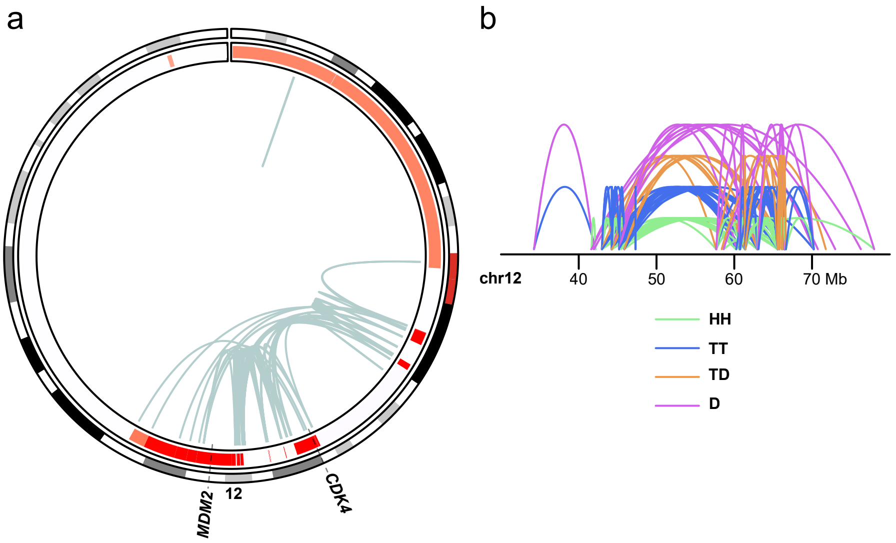

Supplementary Figure 3. Combined copy number and structural variant data for Case 4. a) WGS circos plot: Red regions in the inner circular track of the circos plot indicate copy number gains, with higher level amplifications being in a more intense shade and lower-level gains in a lighter shade. Intrachromosomal structural variants are depicted in light blue. Data is based on copy number array and mate pair whole-genome sequencing. **b) Structural variant distribution:** Intrachromosomal structural variants plotted based on read mapping orientation. Abbreviations: HH = head-to-head inversion, TT = tail-to-tail inversion, TD = duplication type and D = deletion type. Mb = mega-base-pair.

Supplementary Figure 4

Case 5a - Low-grade central osteosarcoma

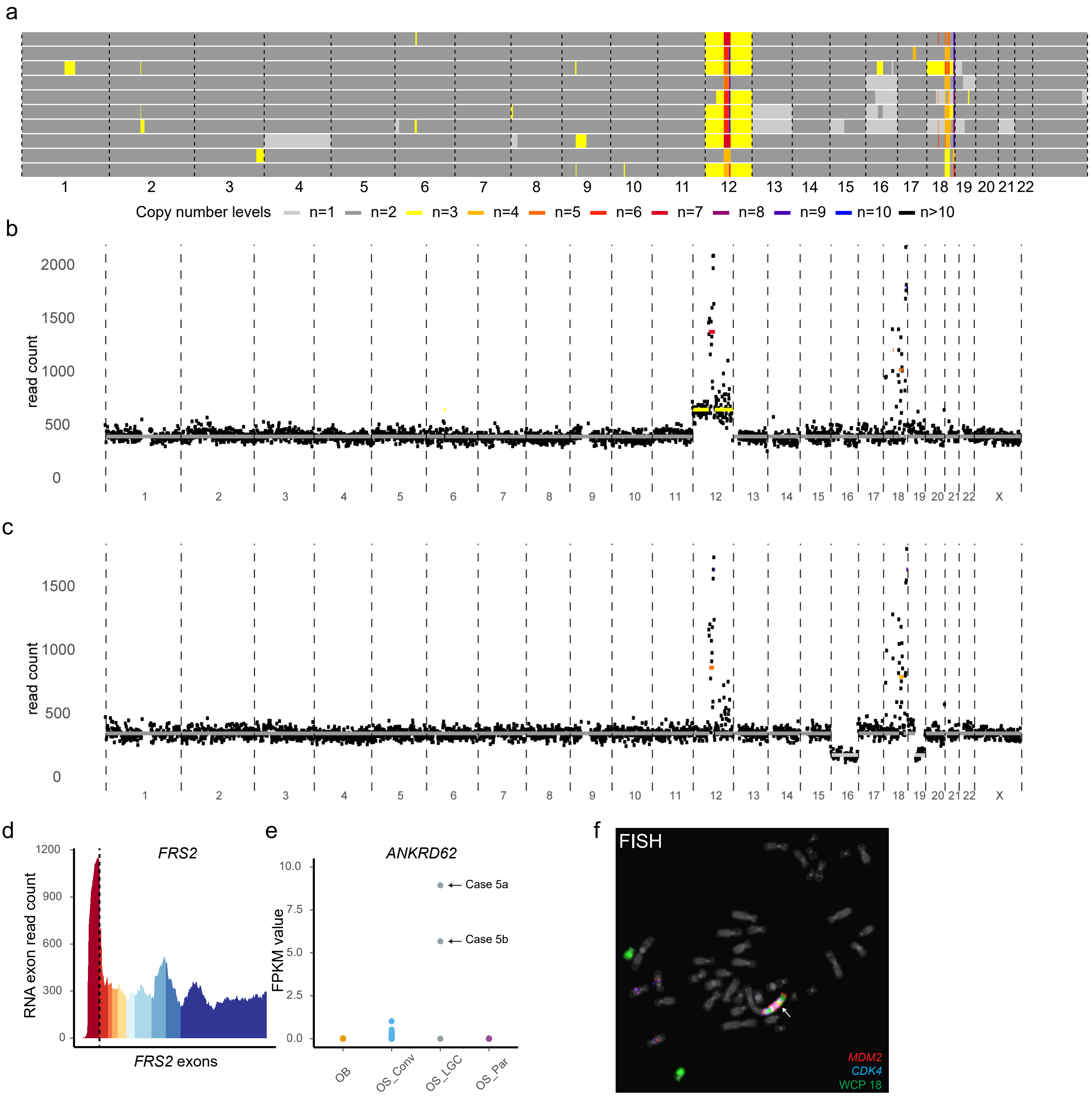

Supplementary Figure 4. Combined copy number, structural variant and transcriptomic data for Case 5a. a) Single cell whole-genome heatmap: Genome-wide copy numbers of sequenced aberrant cells. Each row represents a single cell. A total of 96 individual cells were sequenced, and non-neoplastic cells were excluded from the heatmap. **b-c) Representative single cell:** An example whole-genome copy number view of a single cell. **d) Exon coverage plot:** Read coverage per exon of the given gene. Each exon is depicted in a different colour. The dashed line(s) indicates the breakpoint(s) on the RNA level. **e) Gene expression plot:** Relative gene expression levels of the given gene. The case under study is indicated by an arrow. Abbreviations: OB = osteoblastoma, OS Conv = conventional osteosarcoma, OS LGC = low-grade central osteosarcoma, OS Par = parosteal osteosarcoma (including dedifferentiated parosteal osteosarcoma). **f) FISH:** Fluorescence in situ hybridization (FISH) was conducted using probes targeting specific regions as indicated in the image.

Supplementary Figure 5

Case 5b - Low-grade central osteosarcoma

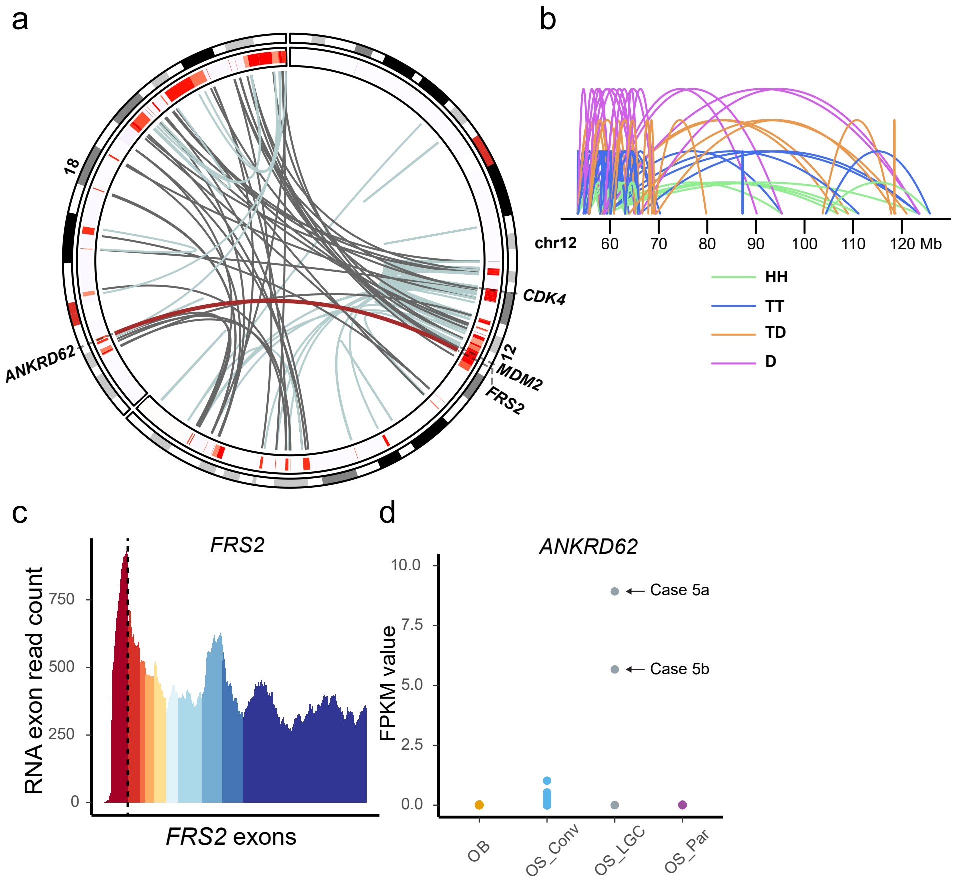

Supplementary Figure 5. Combined copy number, structural variant and transcriptomic data for Case 5b. a) WGS circos plot: Red regions in the inner circular track of the circos plot indicate copy number gains, with higher level amplifications being in a more intense shade and lower-level gains in a lighter shade. Blue regions in the inner circular track of the circos plot indicate copy number losses. Intrachromosomal structural variants are depicted in light blue and interchromosomal structural variants in grey. Selected variants are highlighted in brown. Data is based on copy number array and mate pair whole-genome sequencing. **b) Structural variant distribution:** Intrachromosomal structural variants plotted based on read mapping orientation. Abbreviations: HH = head-to-head inversion, TT = tail-to-tail inversion, TD = duplication type and D = deletion type. Mb = mega-base-pair. **c) Exon coverage plot:** Read coverage per exon of the given gene. Each exon is depicted in a different colour. The dashed line(s) indicates the breakpoint(s) on the RNA level. **d) Gene expression plot:** Relative gene expression levels of the given gene. The case under study is indicated by an arrow. Abbreviations: OB = osteoblastoma, OS Conv = conventional osteosarcoma, OS LGC = low-grade central osteosarcoma, OS Par = parosteal osteosarcoma (including dedifferentiated parosteal osteosarcoma).

Supplementary Figure 6
Case 6 - Parosteal osteosarcoma

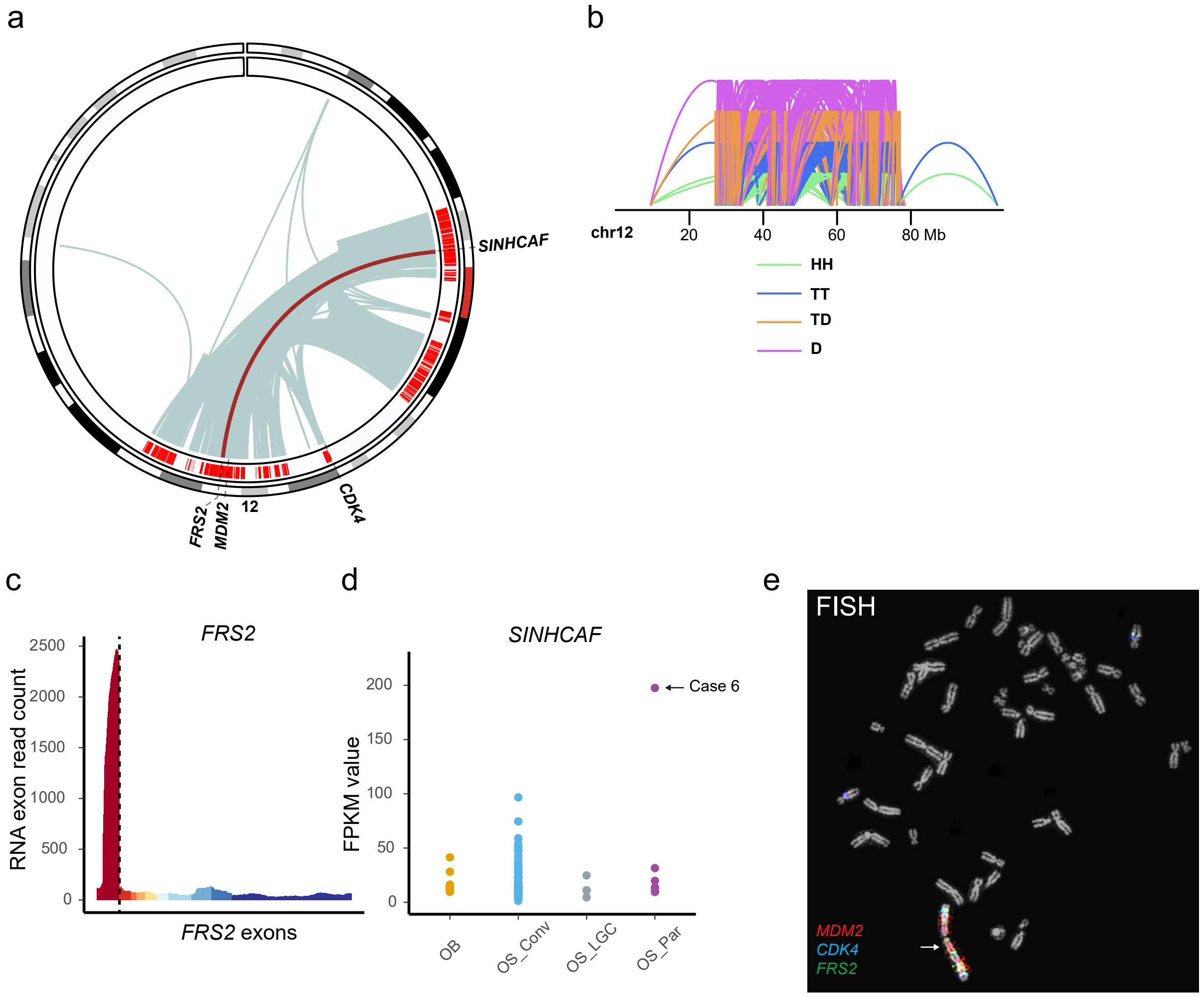

Supplementary Figure 6. Combined copy number, structural variant and transcriptomic data for Case 6. a) WGS circos plot: Red regions in the inner circular track of the circos plot indicate copy number gains, with higher level amplifications being in a more intense shade and lower-level gains in a lighter shade. Blue regions in the inner circular track of the circos plot indicate copy number losses. Intrachromosomal structural variants are depicted in light blue and interchromosomal structural variants in grey. Selected variants are highlighted in brown. Data is based on copy number array and mate pair whole-genome sequencing. **b) Structural variant distribution:** Intrachromosomal structural variants plotted based on read mapping orientation. Abbreviations: HH = head-to-head inversion, TT = tail-to-tail inversion, TD = duplication type and D = deletion type. Mb = mega-base-pair. **c) Exon coverage plot:** Read coverage per exon of the given gene. Each exon is depicted in a different colour. The dashed line(s) indicates the breakpoint(s) on the RNA level. **d) Gene expression plot:** Relative gene expression levels of the given gene. The case under study is indicated by an arrow. Abbreviations: OB = osteoblastoma, OS Conv = conventional osteosarcoma, OS LGC = low-grade central osteosarcoma, OS Par = parosteal osteosarcoma (including dedifferentiated parosteal osteosarcoma). **e) FISH:** Fluorescence in situ hybridization (FISH) was conducted using probes targeting specific regions as indicated in the image.

Supplementary Figure 7

Case 7 - Dedifferentiated parosteal osteosarcoma

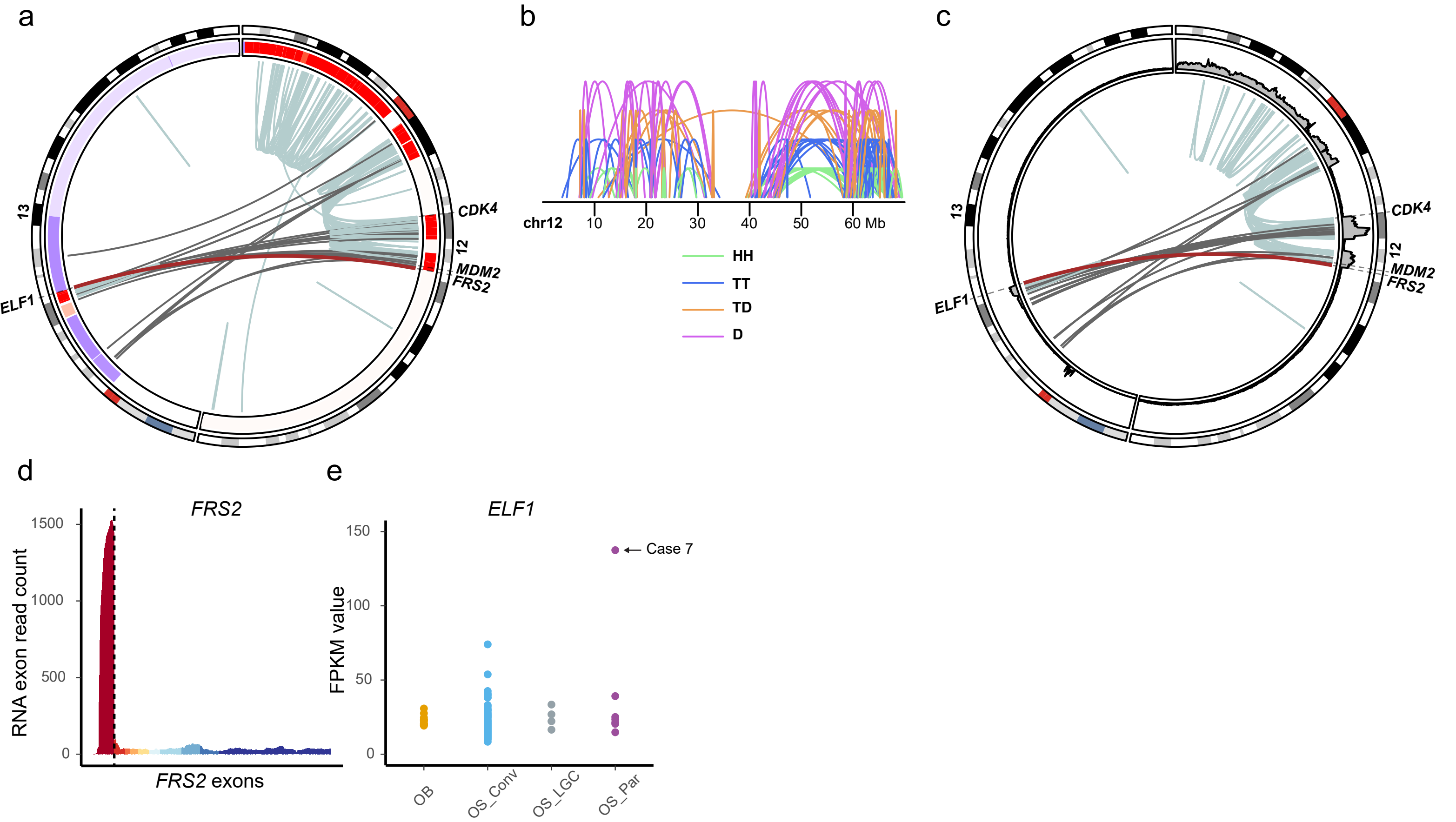

Supplementary Figure 7. Combined copy number, structural variant and transcriptomic data. for Case 7. a) WGS circos plot: Red regions in the inner circular track of the circos plot indicate copy number gains, with higher level amplifications being in a more intense shade and lower-level gains in a lighter shade. Blue regions in the inner circular track of the circos plot indicate copy number losses. Intrachromosomal structural variants are depicted in light blue and interchromosomal structural variants in grey. Selected variants are highlighted in brown. Data is based on copy number array and mate pair whole-genome sequencing. **b) Structural variant distribution:** Intrachromosomal structural variants plotted based on read mapping orientation. Abbreviations: HH = head-to-head inversion, TT = tail-to-tail inversion, TD = duplication type and D = deletion type. Mb = mega-base-pair. **c) Longread WGS circos plot:** Coverage levels are plotted in the inner circular track of the circos plot as a proxy for copy number levels. Intrachromosomal structural variants are depicted in light blue and interchromosomal structural variants in grey. Selected variants are highlighted in brown. Data is based on longread whole-genome sequencing. **d) Exon coverage plot:** Read coverage per exon of the given gene. Each exon is depicted in a different colour. The dashed line(s) indicates the breakpoint(s) on the RNA level. **e) Gene expression plot:** Relative gene expression levels of the given gene. The case under study is indicated by an arrow. Abbreviations: OB = osteoblastoma, OS Conv = conventional osteosarcoma, OS LGC = low-grade central osteosarcoma, OS Par = parosteal osteosarcoma (including dedifferentiated parosteal osteosarcoma).

Supplementary Figure 8

Case 8 - Parosteal osteosarcoma

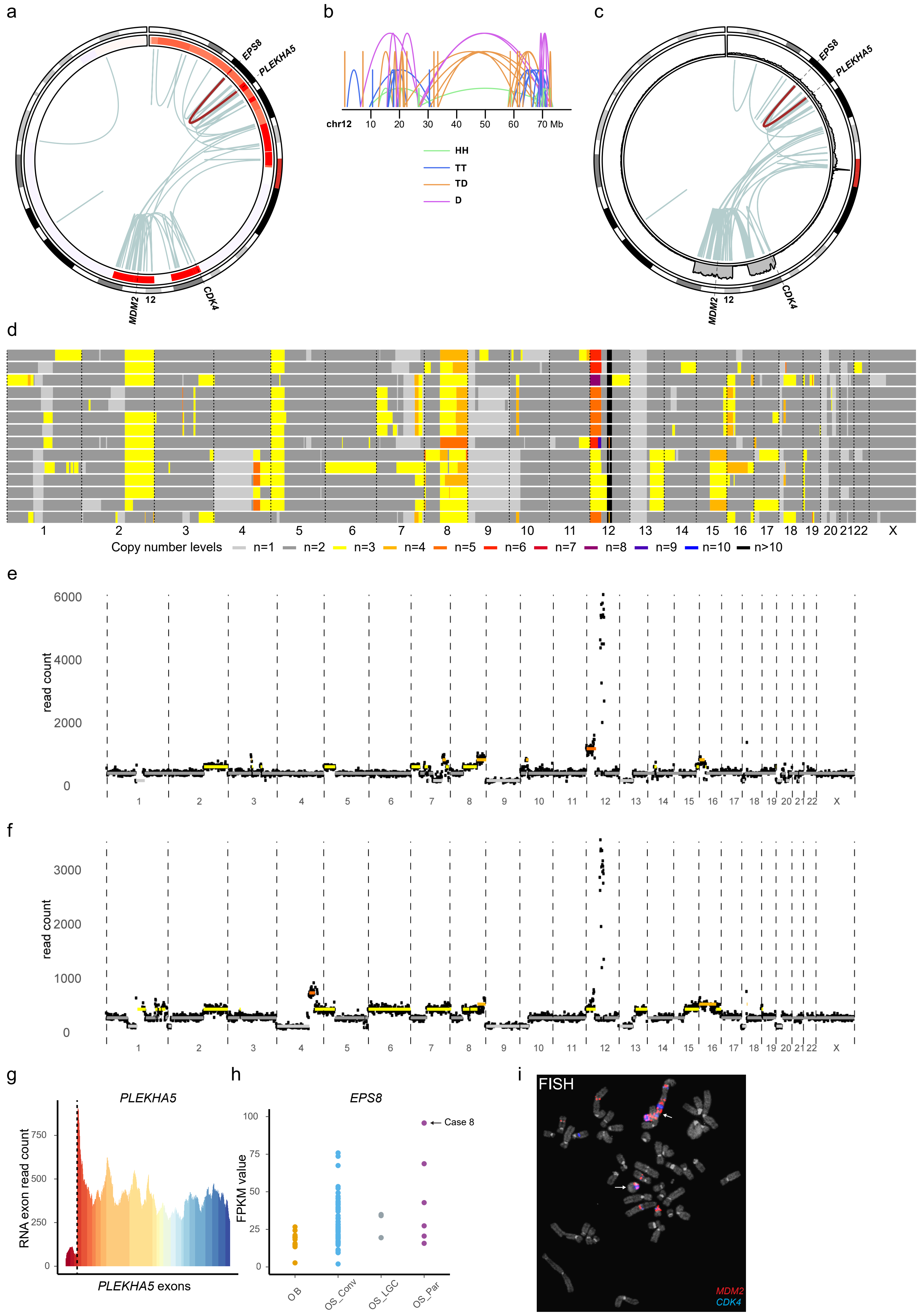

Supplementary Figure 8. Combined copy number, structural variant and transcriptomic data for Case 8. a) WGS circos plot: Red regions in the inner circular track of the circos plot indicate copy number gains, with higher level amplifications being in a more intense shade and lower-level gains in a lighter shade. Blue regions in the inner circular track of the circos plot indicate copy number losses. Intrachromosomal structural variants are depicted in light blue and interchromosomal structural variants in grey. Selected variants are highlighted in brown. Data is based on copy number array and mate pair whole-genome sequencing. **b) Structural variant distribution:** Intrachromosomal structural variants plotted based on read mapping orientation. Abbreviations: HH = head-to-head inversion, TT = tail-to-tail inversion, TD = duplication type and D = deletion type. Mb = mega-base-pair. **c) Longread WGS circos plot:** Coverage levels are plotted in the inner circular track of the circos plot as a proxy for copy number levels. Intrachromosomal structural variants are depicted in light blue and interchromosomal structural variants in grey. Selected variants are highlighted in brown. Data is based on longread whole-genome sequencing. **d) Single cell whole-genome heatmap:** Genome-wide copy numbers of sequenced aberrant cells. Each row represents a single cell. A total of 96 individual cells were sequenced, and non-neoplastic cells were excluded from the heatmap. **e-f) Representative single cell:** An example whole-genome copy number view of a single cell. **g) Exon coverage plot:** Read coverage per exon of the given gene. Each exon is depicted in a different colour. The dashed line(s) indicates the breakpoint(s) on the RNA level. **h) Gene expression plot:** Relative gene expression levels of the given gene. The case under study is indicated by an arrow. Abbreviations: OB = osteoblastoma, OS Conv = conventional osteosarcoma, OS LGC = low-grade central osteosarcoma, OS Par = parosteal osteosarcoma (including dedifferentiated parosteal osteosarcoma). **i) FISH:** Fluorescence in situ hybridization (FISH) was conducted using probes targeting specific regions as indicated in the image.

Supplementary Figure 9

Case 9a - Conventional osteosarcoma

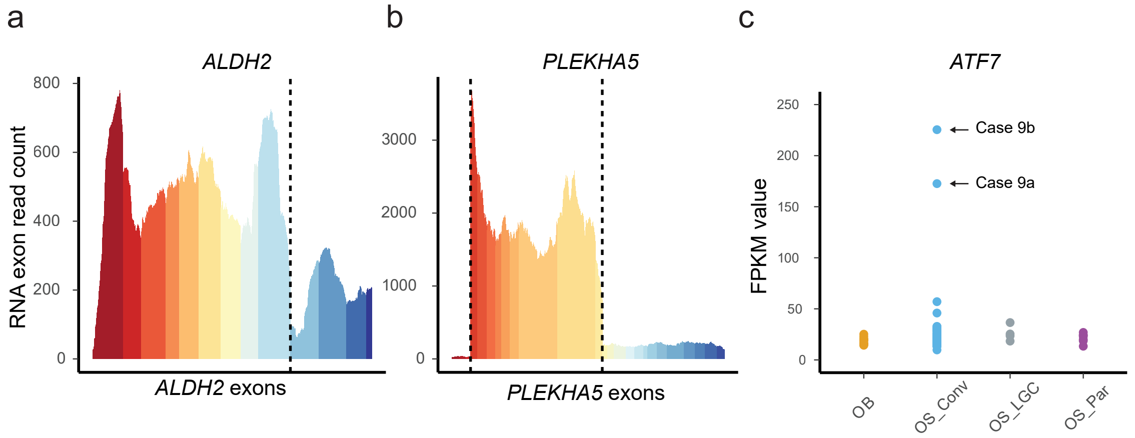

Supplementary Figure 9. Transcriptomic data for Case 9a. a-b) Exon coverage plot: Read coverage per exon of the given gene. Each exon is depicted in a different colour. The dashed line(s) indicates the break-point(s) on the RNA level. **c) Gene expression plot:** Relative gene expression levels of the given gene. The case under study is indicated by an arrow. Abbreviations: OB = osteoblastoma, OS Conv = conventional osteosarcoma, OS LGC = low-grade central osteosarcoma, OS Par = parosteal osteosarcoma (including dedifferentiated parosteal osteosarcoma).

Supplementary Figure 10

Case 9b - Conventional osteosarcoma

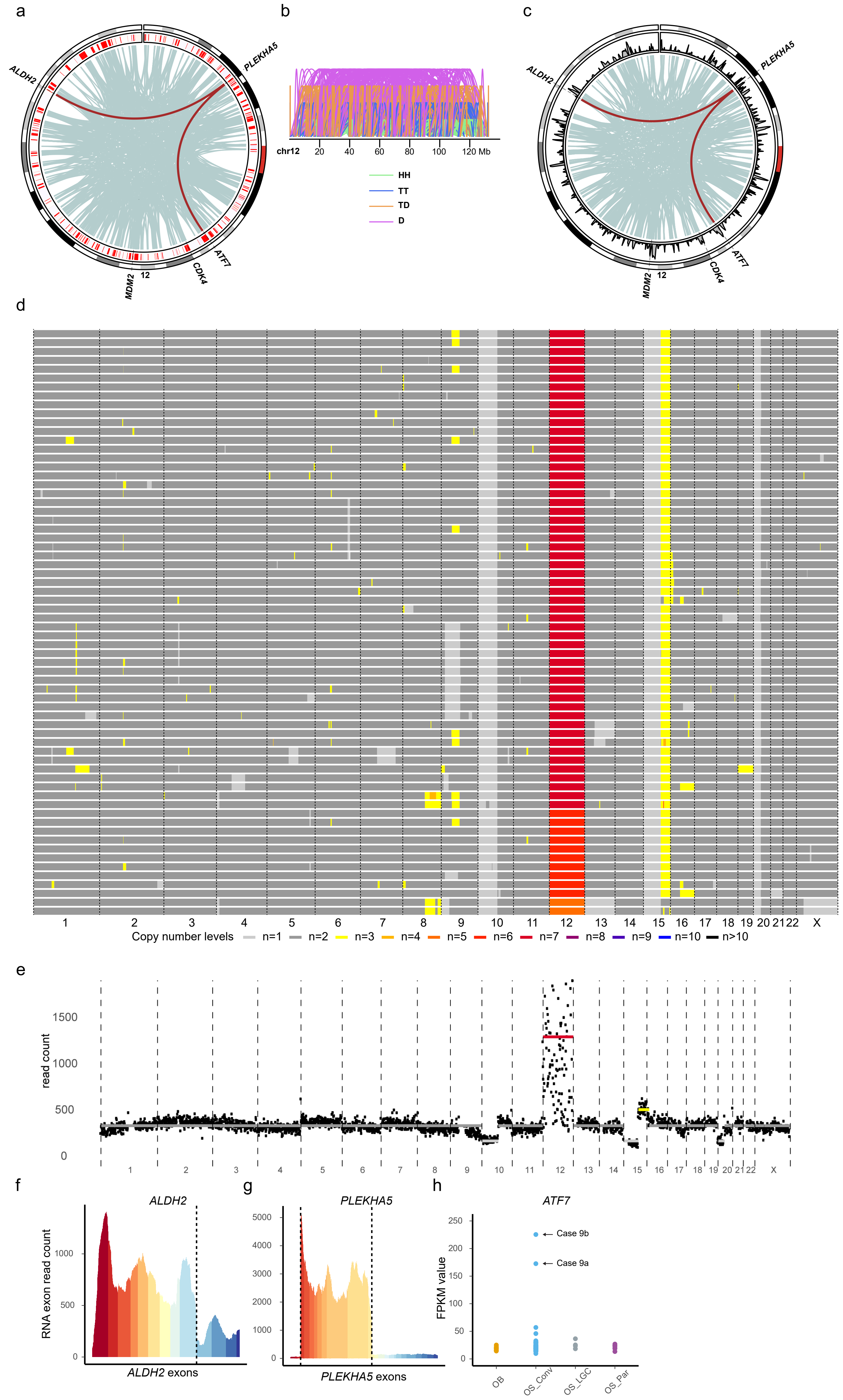

Supplementary Figure 10. Combined copy number, structural variant and transcriptomic data for Case 9b. a) WGS circos plot: Red regions in the inner circular track of the circos plot indicate copy number gains, with higher level amplifications being in a more intense shade and lower-level gains in a lighter shade. Blue regions in the inner circular track of the circos plot indicate copy number losses. Intrachromosomal structural variants are depicted in light blue and interchromosomal structural variants in grey. Selected variants are highlighted in brown. Data is based on copy number array and mate pair whole-genome sequencing. **b) Structural variant distribution:** Intrachromosomal structural variants plotted based on read mapping orientation. Abbreviations: HH = head-to-head inversion, TT = tail-to-tail inversion, TD = duplication type and D = deletion type. Mb = mega-base-pair. **c) Longread WGS circos plot:** Coverage levels are plotted in the inner circular track of the circos plot as a proxy for copy number levels. Intrachromosomal structural variants are depicted in light blue and interchromosomal structural variants in grey. Selected variants are highlighted in brown. Data is based on longread whole-genome sequencing. **d) Single cell whole-genome heatmap:** Genome-wide copy numbers of sequenced aberrant cells. Each row represents a single cell. A total of 96 individual cells were sequenced, and non-neoplastic cells were excluded from the heatmap. **e) Representative single cell:** An example whole-genome copy number view of a single cell. **f-g) Exon coverage plot:** Read coverage per exon of the given gene. Each exon is depicted in a different colour. The dashed line(s) indicates the breakpoint(s) on the RNA level. **h) Gene expression plot:** Relative gene expression levels of the given gene. The case under study is indicated by an arrow. Abbreviations: OB = osteoblastoma, OS Conv = conventional osteosarcoma, OS LGC = low-grade central osteosarcoma, OS Par = parosteal osteosarcoma (including dedifferentiated parosteal osteosarcoma).

Supplementary Figure 11
Case 10 - Dedifferentiated parosteal osteosarcoma

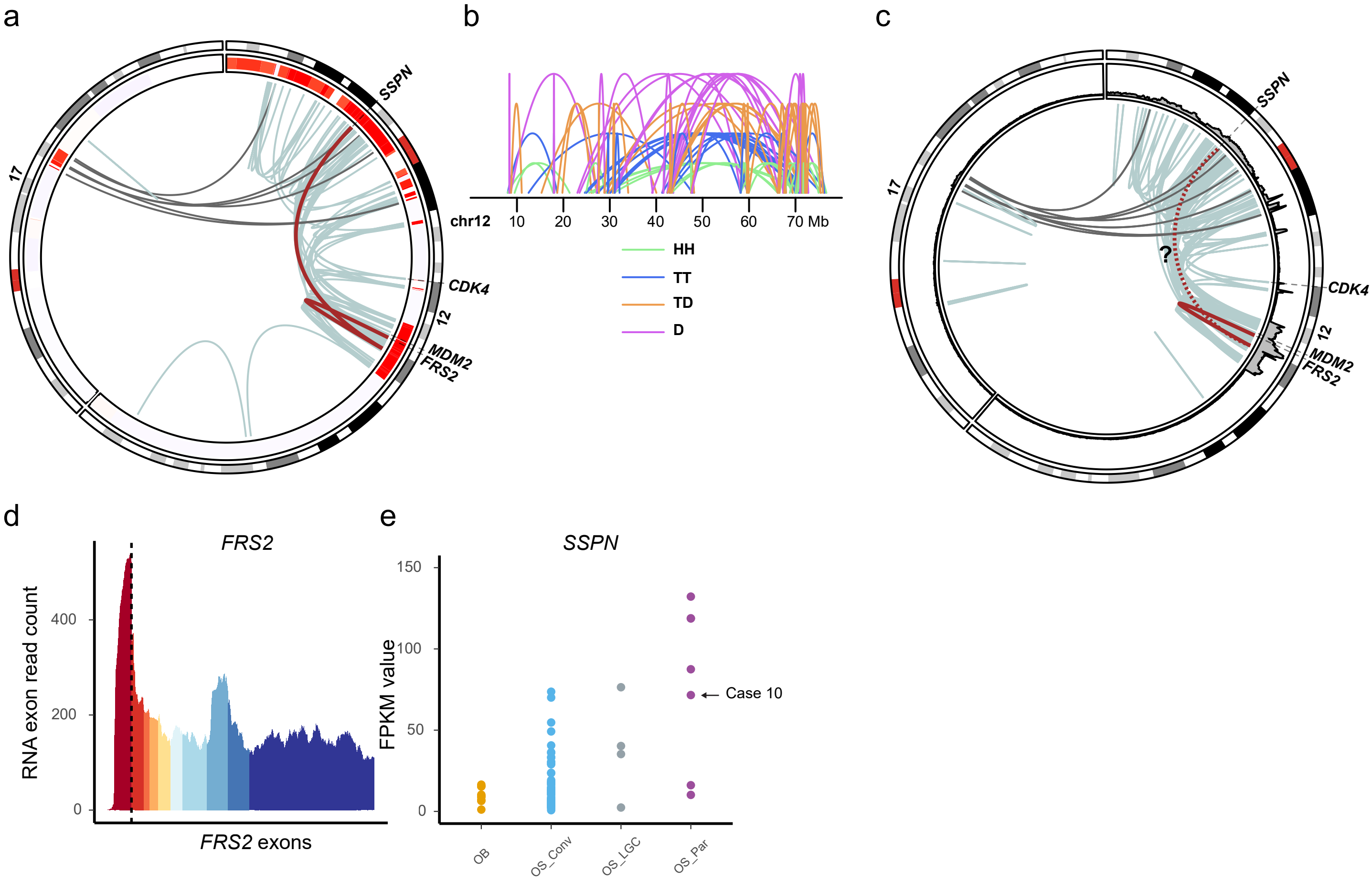

Supplementary Figure 11. Combined copy number, structural variant and transcriptomic data for Case 10. a) WGS circos plot: Red regions in the inner circular track of the circos plot indicate copy number gains, with higher level amplifications being in a more intense shade and lower-level gains in a lighter shade. Blue regions in the inner circular track of the circos plot indicate copy number losses. Intrachromosomal structural variants are depicted in light blue and interchromosomal structural variants in grey. Selected variants are highlighted in brown. Data is based on copy number array and mate pair whole-genome sequencing. **b) Structural variant distribution:** Intrachromosomal structural variants plotted based on read mapping orientation. Abbreviations: HH = head-to-head inversion, TT = tail-to-tail inversion, TD = duplication type and D = deletion type. Mb = mega-base-pair. **c) Longread WGS circos plot:** Coverage levels are plotted in the inner circular track of the circos plot as a proxy for copy number levels. Intrachromosomal structural variants are depicted in light blue and interchromosomal structural variants in grey. Selected variants are highlighted in brown. Data is based on longread whole-genome sequencing. **d) Exon coverage plot:** Read coverage per exon of the given gene. Each exon is depicted in a different colour. The dashed line(s) indicates the breakpoint(s) on the RNA level. **e) Gene expression plot:** Relative gene expression levels of the given gene. The case under study is indicated by an arrow. Abbreviations: OB = osteoblastoma, OS Conv = conventional osteosarcoma, OS LGC = low-grade central osteosarcoma, OS Par = parosteal osteosarcoma (including dedifferentiated parosteal osteosarcoma).

Supplementary Figure 12

OS191 - Conventional osteosarcoma

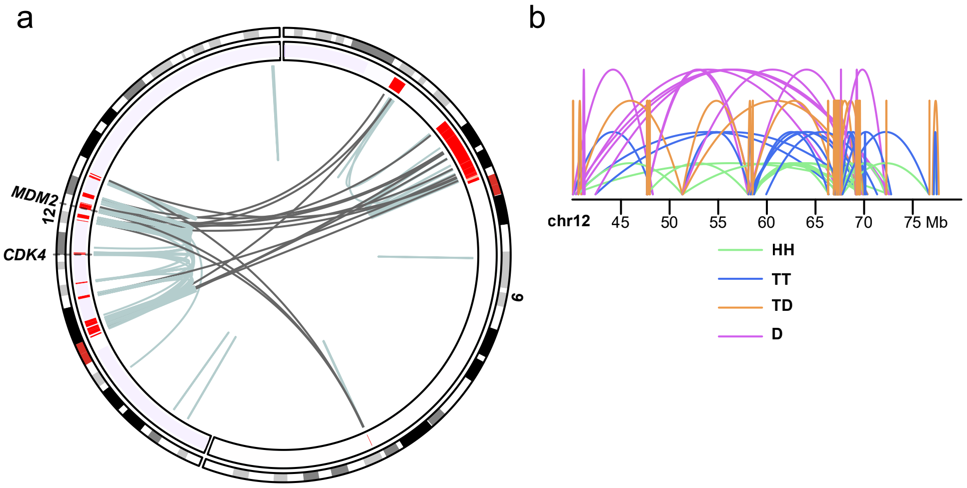

Supplementary Figure 12. Combined copy number and structural variant data for OS191. a) WGS circos plot: Red regions in the inner circular track of the circos plot indicate copy number gains, with higher level amplifications being in a more intense shade and lower-level gains in a lighter shade. Blue regions in the inner circular track of the circos plot indicate copy number losses. Intrachromosomal structural variants are depicted in light blue and interchromosomal structural variants in grey. Selected variants are highlighted in brown. Data is based on copy number array and mate pair whole-genome sequencing. **b) Structural variant distribution:** Intrachromosomal structural variants plotted based on read mapping orientation. Abbreviations: HH = head-to-head inversion, TT = tail-to-tail inversion, TD = duplication type and D = deletion type. Mb = mega-base-pair.

Supplementary Figure 13

Case 12 - Dedifferentiated parosteal osteosarcoma

Case 12a: Case 12c:

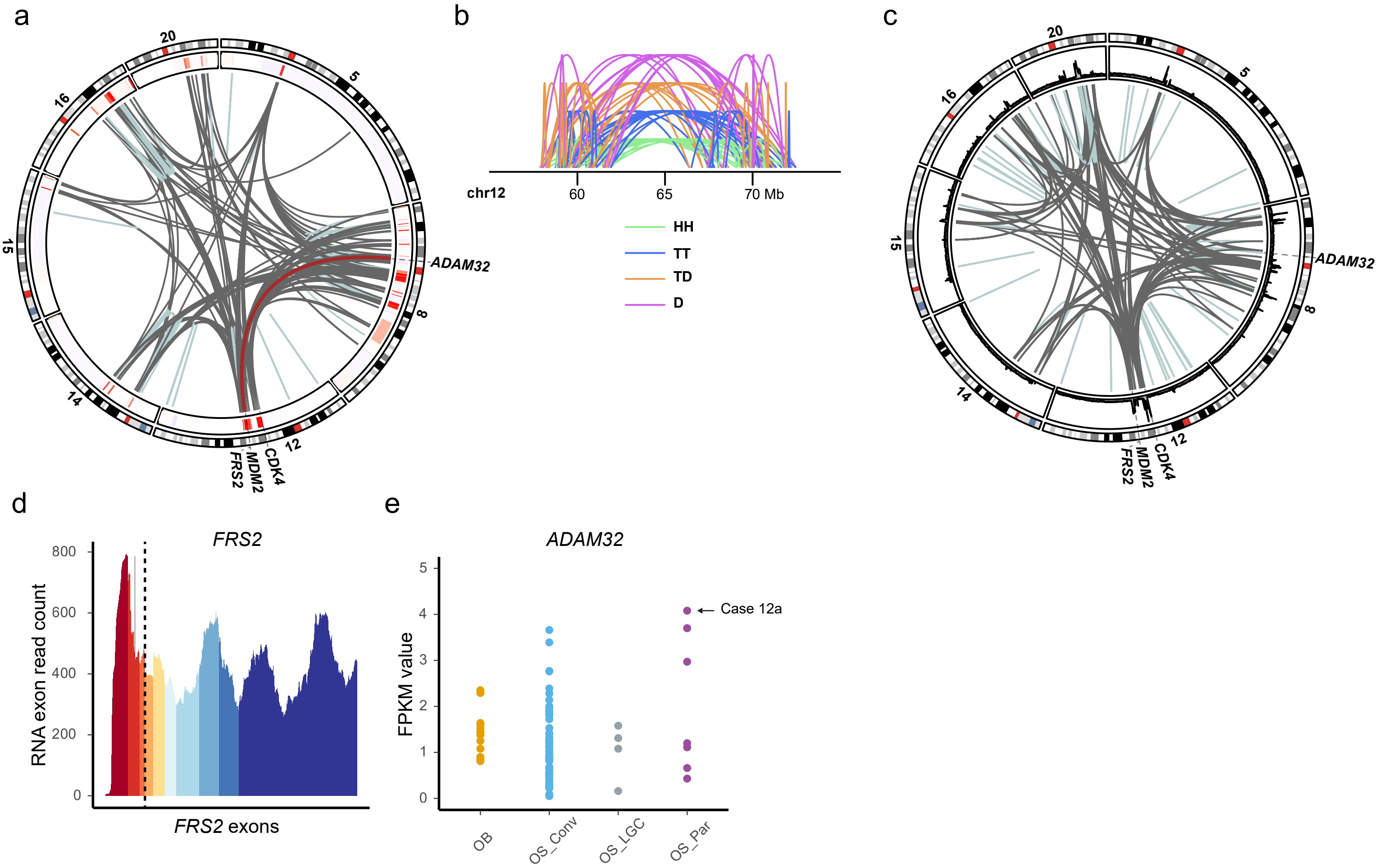

Supplementary Figure 13. Combined copy number, structural variant and transcriptomic data for Case 12. a) WGS circos plot: Red regions in the inner circular track of the circos plot indicate copy number gains, with higher level amplifications being in a more intense shade and lower-level gains in a lighter shade. Blue regions in the inner circular track of the circos plot indicate copy number losses. Intrachromosomal structural variants are depicted in light blue and interchromosomal structural variants in grey. Selected variants are highlighted in brown. Data is based on copy number array and mate pair whole-genome sequencing of Case 12a. **b) Structural variant distribution:** Intrachromosomal structural variants plotted based on read mapping orientation. Abbreviations: HH = head-to-head inversion, TT = tail-to-tail inversion, TD = duplication type and D = deletion type. Mb = mega-base-pair. **c) Longread WGS circos plot:** Coverage levels are plotted in the inner circular track of the circos plot as a proxy for copy number levels. Intrachromosomal structural variants are depicted in light blue and interchromosomal structural variants in grey. Data is based on longread whole-genome sequencing of Case 12c. **d) Exon coverage plot:** Read coverage per exon of the given gene. Each exon is depicted in a different colour. The dashed line(s) indicates the breakpoint(s) on the RNA level. **e) Gene expression plot:** Relative gene expression levels of the given gene. The case under study is indicated by an arrow. Abbreviations: OB = osteoblastoma, OS Conv = conventional osteosarcoma, OS LGC = low-grade central osteosarcoma, OS Par = parosteal osteosarcoma (including dedifferentiated parosteal osteosarcoma).

Supplementary Figure 14

Case 13 - Conventional osteosarcoma

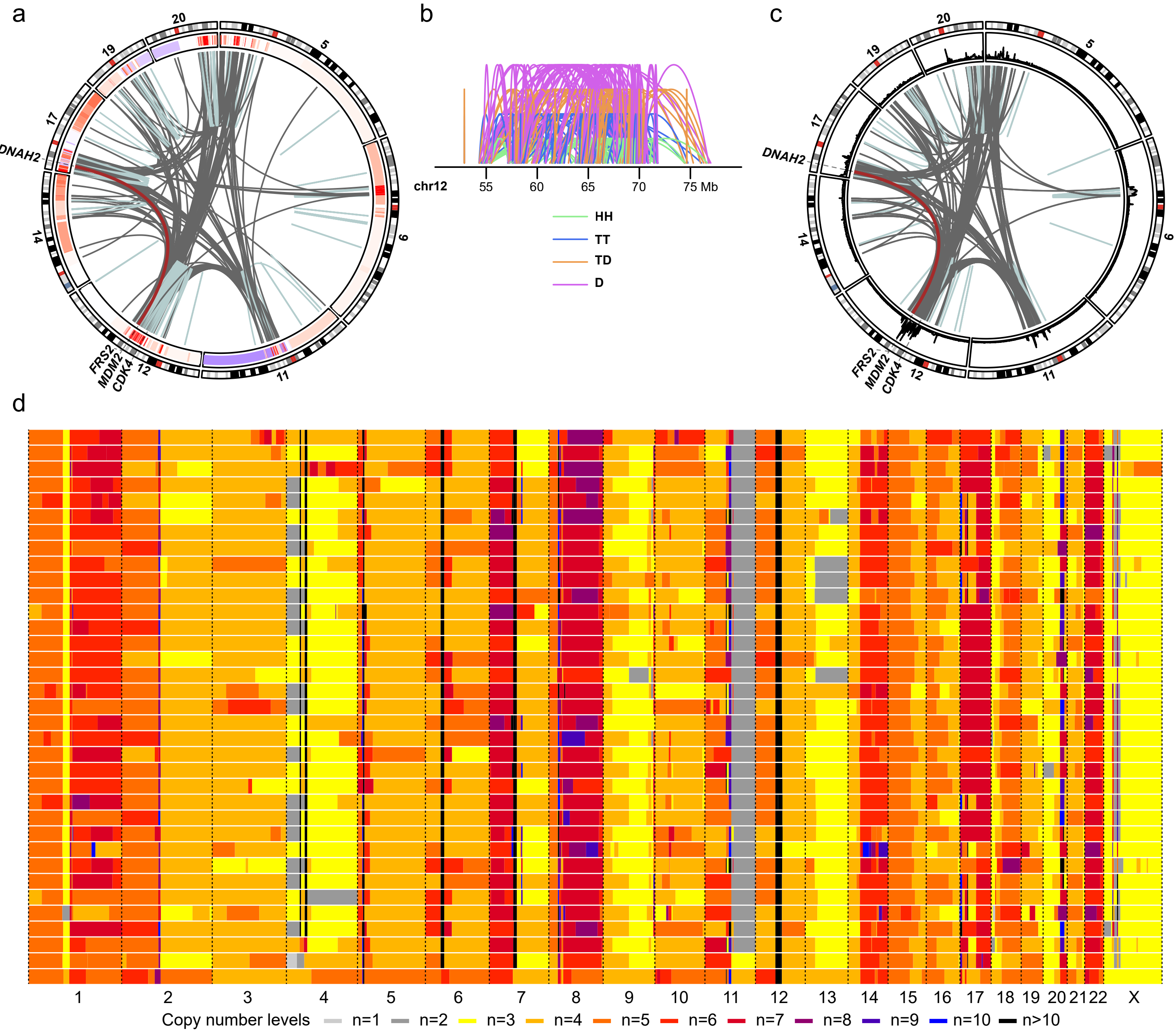

Supplementary Figure 14. Combined copy number and structural variant data for Case 13. a) WGS circos plot: Red regions in the inner circular track of the circos plot indicate copy number gains, with higher level amplifications being in a more intense shade and lower-level gains in a lighter shade. Blue regions in the inner circular track of the circos plot indicate copy number losses. Intrachromosomal structural variants are depicted in light blue and interchromosomal structural variants in grey. Selected variants affecting *FRS2* are highlighted in brown. Data is based on copy number array and mate pair whole-genome sequencing. **b) Structural variant distribution:** Intrachromosomal structural variants plotted based on read mapping orientation. Abbreviations: HH = head-to-head inversion, TT = tail-to-tail inversion, TD = duplication type and D = deletion type. Mb = mega-base-pair. **c) Longread WGS circos plot:** Coverage levels are plotted in the inner circular track of the circos plot as a proxy for copy number levels. Intrachromosomal structural variants are depicted in light blue and interchromosomal structural variants in grey. Selected variants affecting *FRS2* are highlighted in brown. Data is based on longread whole-genome sequencing. **d) Single cell whole-genome heatmap:** Genome-wide copy numbers of sequenced aberrant cells. Each row represents a single cell. A total of 48 individual cells were sequenced, and non-neoplastic cells were excluded from the heatmap.

Supplementary Figure 15

Case 14 - Conventional osteosarcoma

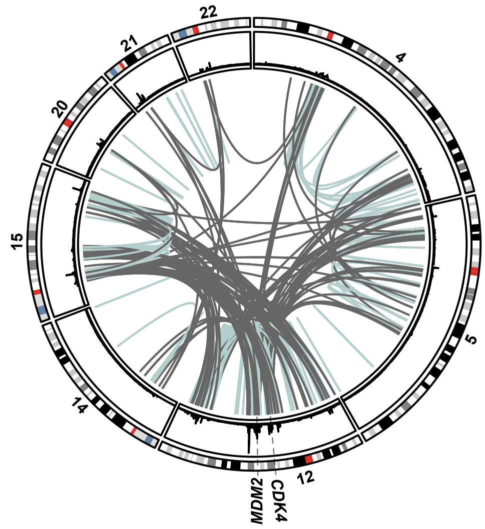

Supplementary Figure 15. Combined copy number and structural variant data for Case 14. Longread WGS circos plot: Coverage levels are plotted in the inner circular track of the circos plot as a proxy for copy number levels. Intrachromosomal structural variants are depicted in light blue and interchromosomal structural variants in grey. Data is based on longread whole-genome sequencing.

Supplementary Figure 16

Case 15 - Conventional osteosarcoma

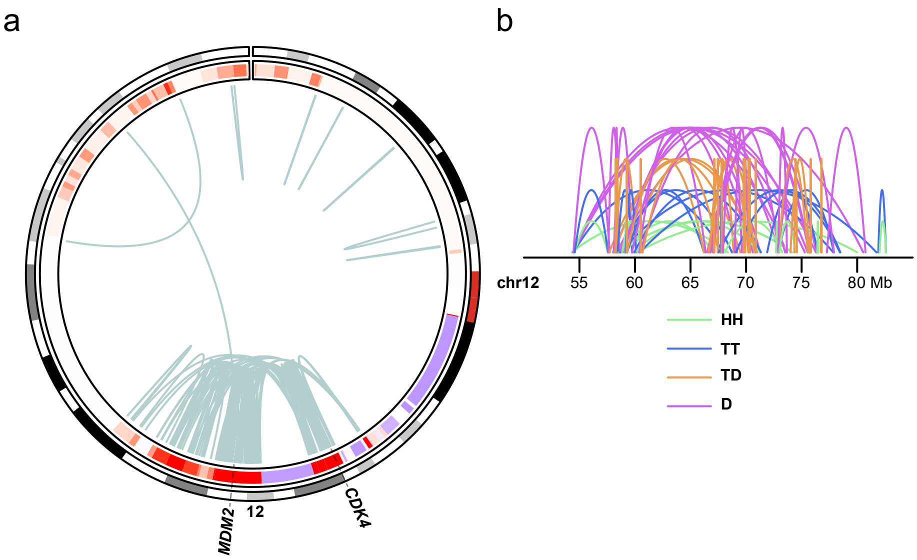

Supplementary Figure 16. Combined copy number and structural variant data for Case 15. a) WGS circos plot: Red regions in the inner circular track of the circos plot indicate copy number gains, with higher level amplifications being in a more intense shade and lower-level gains in a lighter shade. Blue regions in the inner circular track of the circos plot indicate copy number losses. Intrachromosomal structural variants are depicted in light blue and interchromosomal structural variants in grey. Data is based on copy number array and mate pair whole-genome sequencing. **b) Structural variant distribution:** Intrachromosomal structural variants plotted based on read mapping orientation. Abbreviations: HH = head-to-head inversion, TT = tail-to-tail inversion, TD = duplication type and D = deletion type. Mb = mega-base-pair.

Supplementary Figure 17

Case 16 - Conventional osteosarcoma

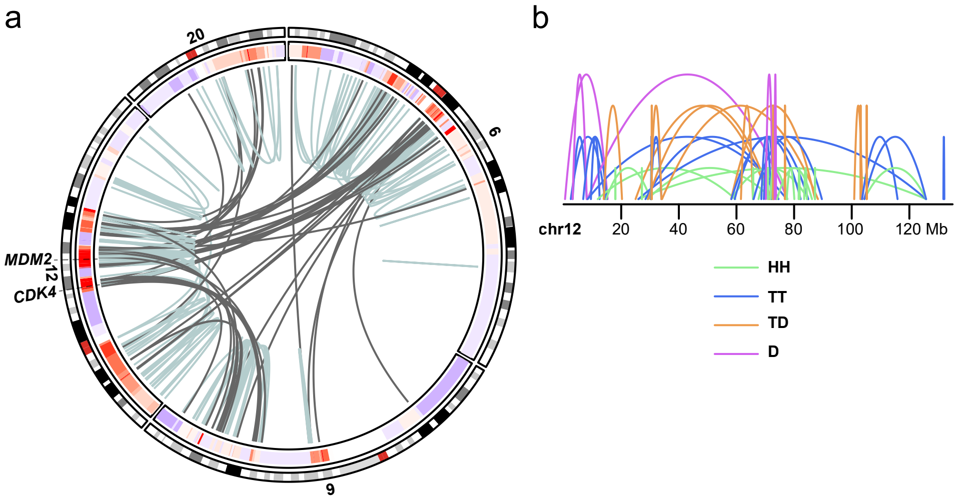

Supplementary Figure 17. Combined copy number and structural variant data for Case 16. a) WGS circos plot: Red regions in the inner circular track of the circos plot indicate copy number gains, with higher level amplifications being in a more intense shade and lower-level gains in a lighter shade. Blue regions in the inner circular track of the circos plot indicate copy number losses. Intrachromosomal structural variants are depicted in light blue and interchromosomal structural variants in grey. Data is based on copy number array and mate pair whole-genome sequencing. **b) Structural variant distribution:** Intrachromosomal structural variants plotted based on read mapping orientation. Abbreviations: HH = head-to-head inversion, TT = tail-to-tail inversion, TD = duplication type and D = deletion type. Mb = mega-base-pair.

Supplementary Figure 18

OS131 - Conventional osteosarcoma

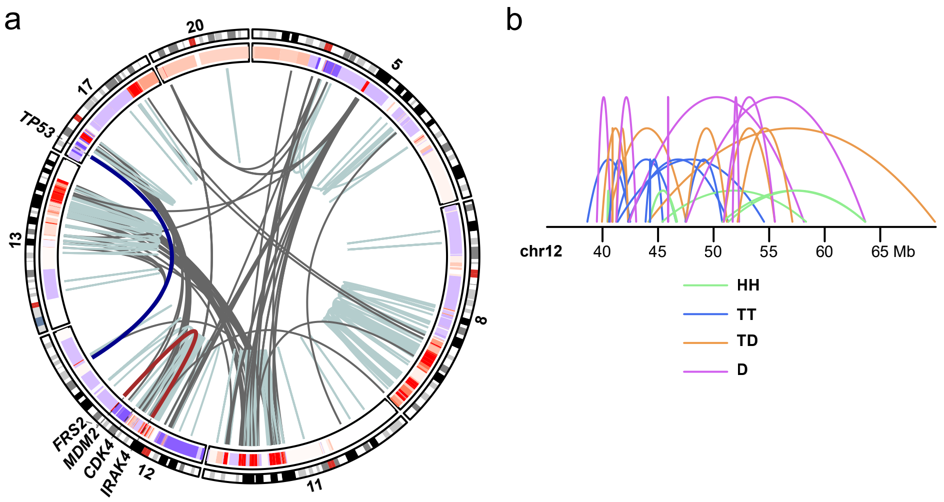

Supplementary Figure 18. Combined copy number and structural variant data for OS131. a) WGS circos plot: Red regions in the inner circular track of the circos plot indicate copy number gains, with higher level amplifications being in a more intense shade and lower-level gains in a lighter shade. Blue regions in the inner circular track of the circos plot indicate copy number losses. Intrachromosomal structural variants are depicted in light blue and interchromosomal structural variants in grey. Selected variants affecting *FRS2* and *TP53* are highlighted in brown and blue, respectively. Data is based on copy number array and mate pair whole-genome sequencing. **b) Structural variant distribution:** Intrachromosomal structural variants plotted based on read mapping orientation. Abbreviations: HH = head-to-head inversion, TT = tail-to-tail inversion, TD = duplication type and D = deletion type. Mb = mega-base-pair.

Supplementary Figure 19

Case 17 - Conventional osteosarcoma

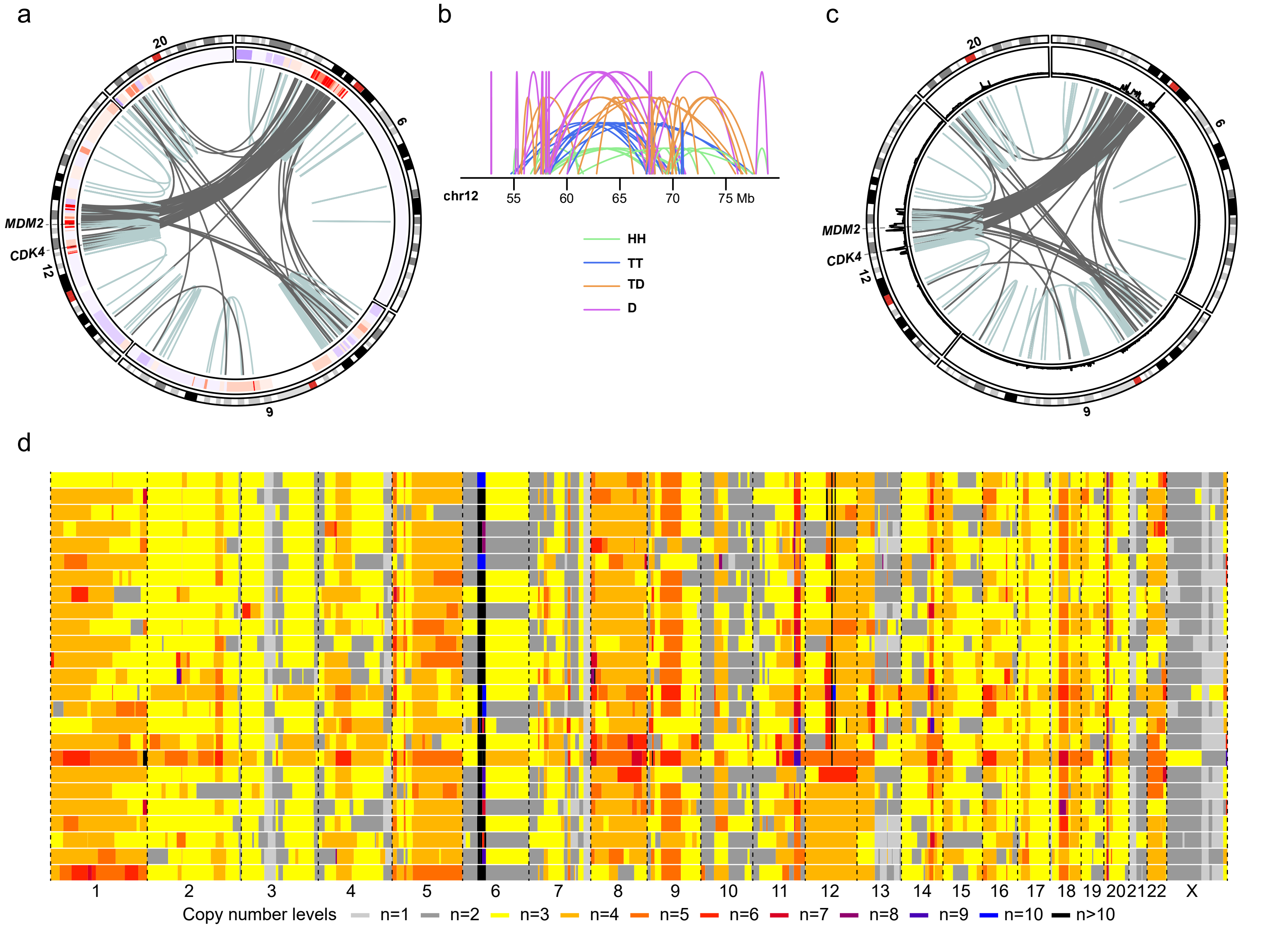

Supplementary Figure 19. Combined copy number and structural variant data for Case 17. a) WGS circos plot: Red regions in the inner circular track of the circos plot indicate copy number gains, with higher level amplifications being in a more intense shade and lower-level gains in a lighter shade. Blue regions in the inner circular track of the circos plot indicate copy number losses. Intrachromosomal structural variants are depicted in light blue and interchromosomal structural variants in grey. Data is based on copy number array and mate pair whole-genome sequencing. **b) Structural variant distribution:** Intrachromosomal structural variants plotted based on read mapping orientation. Abbreviations: HH = head-to-head inversion, TT = tail-to-tail inversion, TD = duplication type and D = deletion type. Mb = mega-base-pair. **c) Longread WGS circos plot:** Coverage levels are plotted in the inner circular track of the circos plot as a proxy for copy number levels. Intrachromosomal structural variants are depicted in light blue and interchromosomal structural variants in grey. **d) Single cell whole-genome heatmap:** Genome-wide copy numbers of sequenced aberrant cells. Each row represents a single cell. A total of 48 individual cells were sequenced, and non-neoplastic cells were excluded from the heatmap.

Supplementary Figure 20
OS061 - Conventional osteosarcoma

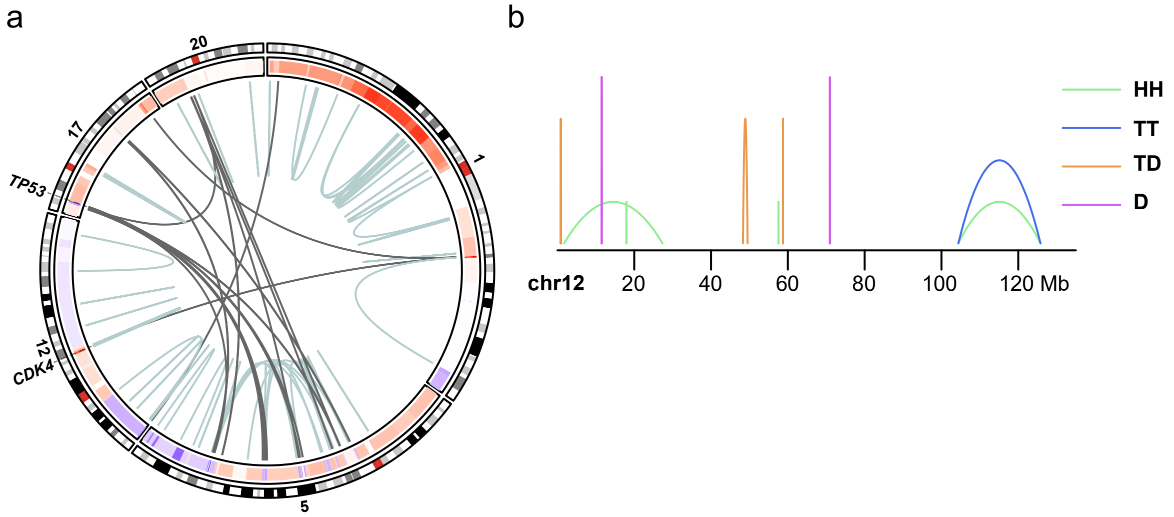

Supplementary Figure 20. Combined copy number and structural variant data for OS061. a) WGS circos plot: Red regions in the inner circular track of the circos plot indicate copy number gains, with higher level amplifications being in a more intense shade and lower-level gains in a lighter shade. Blue regions in the inner circular track of the circos plot indicate copy number losses. Intrachromosomal structural variants are depicted in light blue and interchromosomal structural variants in grey. Data is based on copy number array and mate pair whole-genome sequencing. **b) Structural variant distribution:** Intrachromosomal structural variants plotted based on read mapping orientation. Abbreviations: HH = head-to-head inversion, TT = tail-to-tail inversion, TD = duplication type and D = deletion type. Mb = mega-base-pair.

Supplementary Figure 21

OS222 - Conventional osteosarcoma

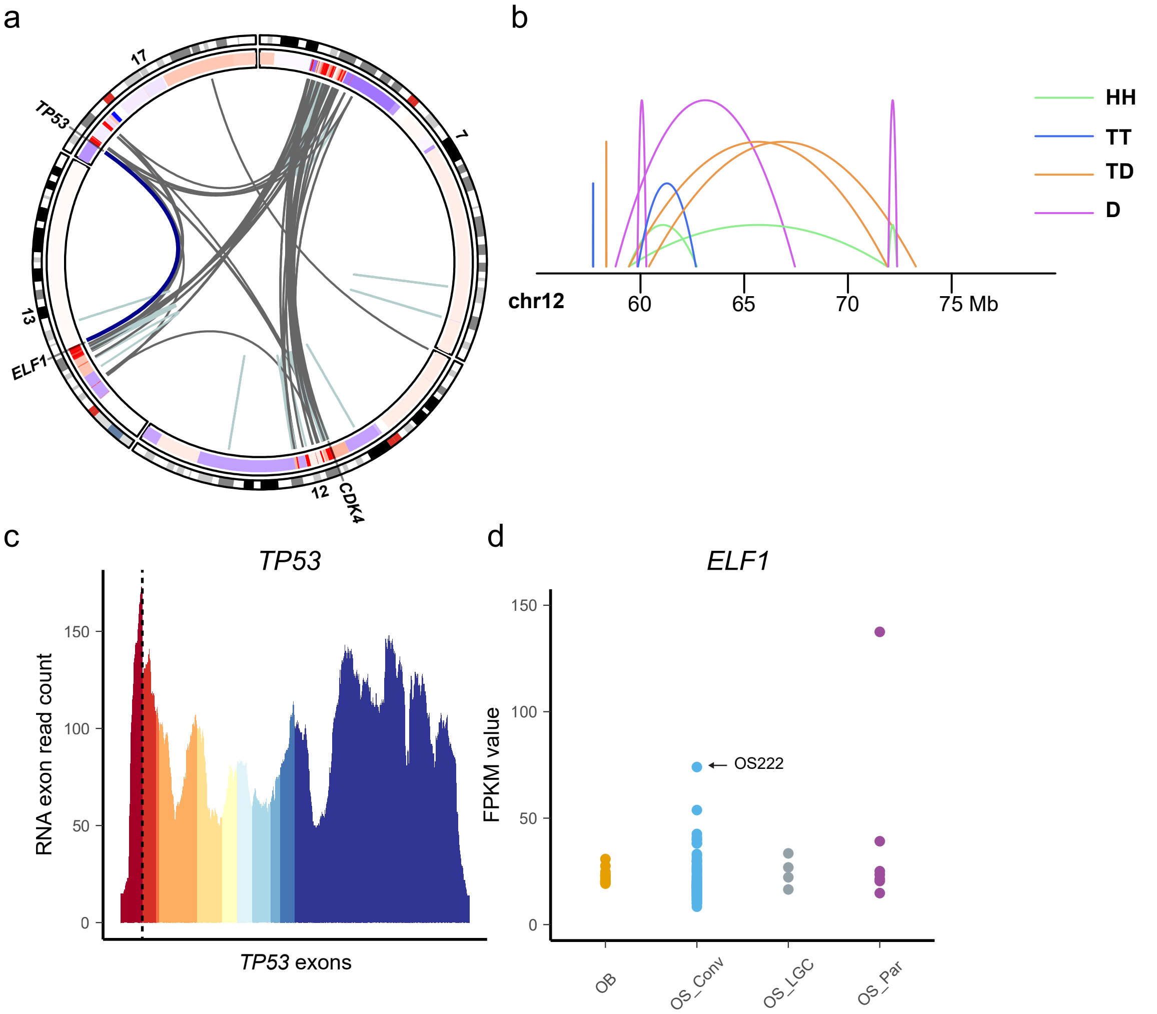

Supplementary Figure 21. Combined copy number, structural variant and transcriptomic data for OS222. a) WGS circos plot: Red regions in the inner circular track of the circos plot indicate copy number gains, with higher level amplifications being in a more intense shade and lower-level gains in a lighter shade. Blue regions in the inner circular track of the circos plot indicate copy number losses. Intrachromosomal structural variants are depicted in light blue and interchromosomal structural variants in grey. Selected variants are highlighted in dark blue. Data is based on copy number array and mate pair whole-genome sequencing. **b) Structural variant distribution:** Intrachromosomal structural variants plotted based on read mapping orientation. Abbreviations: HH = head-to-head inversion, TT = tail-to-tail inversion, TD = duplication type and D = deletion type. Mb = mega-base-pair. **c) Exon coverage plot:** Read coverage per exon of the given gene. Each exon is depicted in a different colour. The dashed line indicates the breakpoint on the RNA level. **d) Gene expression plot:** Relative gene expression levels of the given gene. The case under study is indicated by an arrow. Abbreviations: OB = osteoblastoma, OS Conv = conventional osteosarcoma, OS LGC = low-grade central osteosarcoma, OS Par = parosteal osteosarcoma (including dedifferentiated parosteal osteosarcoma).

Supplementary Figure 22

OS046 - Conventional osteosarcoma

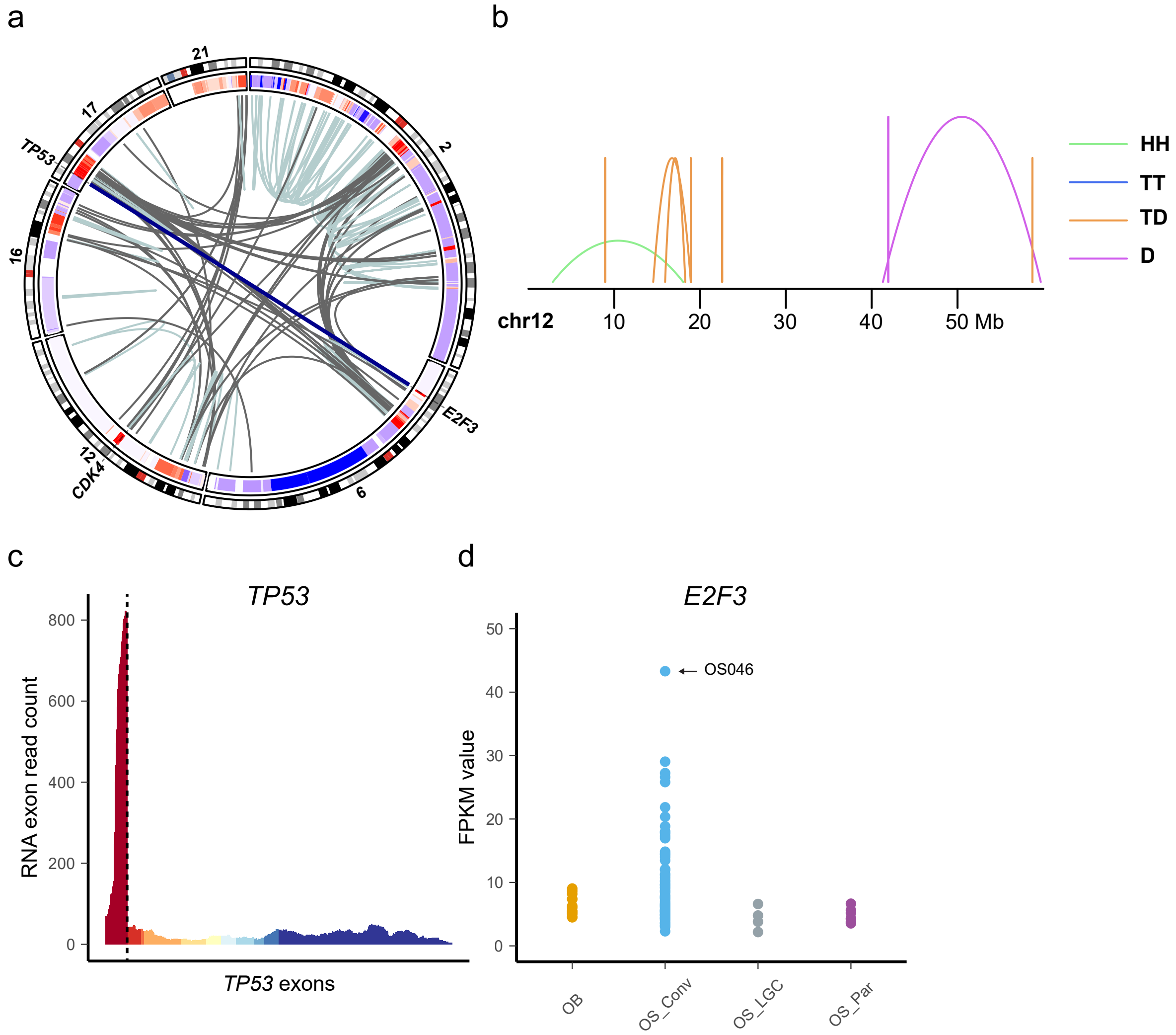

Supplementary Figure 22. Combined copy number, structural variant and transcriptomic data for OS046. a) WGS circos plot: Red regions in the inner circular track of the circos plot indicate copy number gains, with higher level amplifications being in a more intense shade and lower-level gains in a lighter shade. Blue regions in the inner circular track of the circos plot indicate copy number losses. Intrachromosomal structural variants are depicted in light blue and interchromosomal structural variants in grey. Selected variants are highlighted in dark blue. Data is based on copy number array and mate pair whole-genome sequencing. **b) Structural variant distribution:** Intrachromosomal structural variants plotted based on read mapping orientation. Abbreviations: HH = head-to-head inversion, TT = tail-to-tail inversion, TD = duplication type and D = deletion type. Mb = mega-base-pair. **c) Exon coverage plot:** Read coverage per exon of the given gene. Each exon is depicted in a different colour. The dashed line indicates the breakpoint on the RNA level. **d) Gene expression plot:** Relative gene expression levels of the given gene. The case under study is indicated by an arrow. Abbreviations: OB = osteoblastoma, OS Conv = conventional osteosarcoma, OS LGC = low-grade central osteosarcoma, OS Par = parosteal osteosarcoma (including dedifferentiated parosteal osteosarcoma).

Supplementary Figure 23

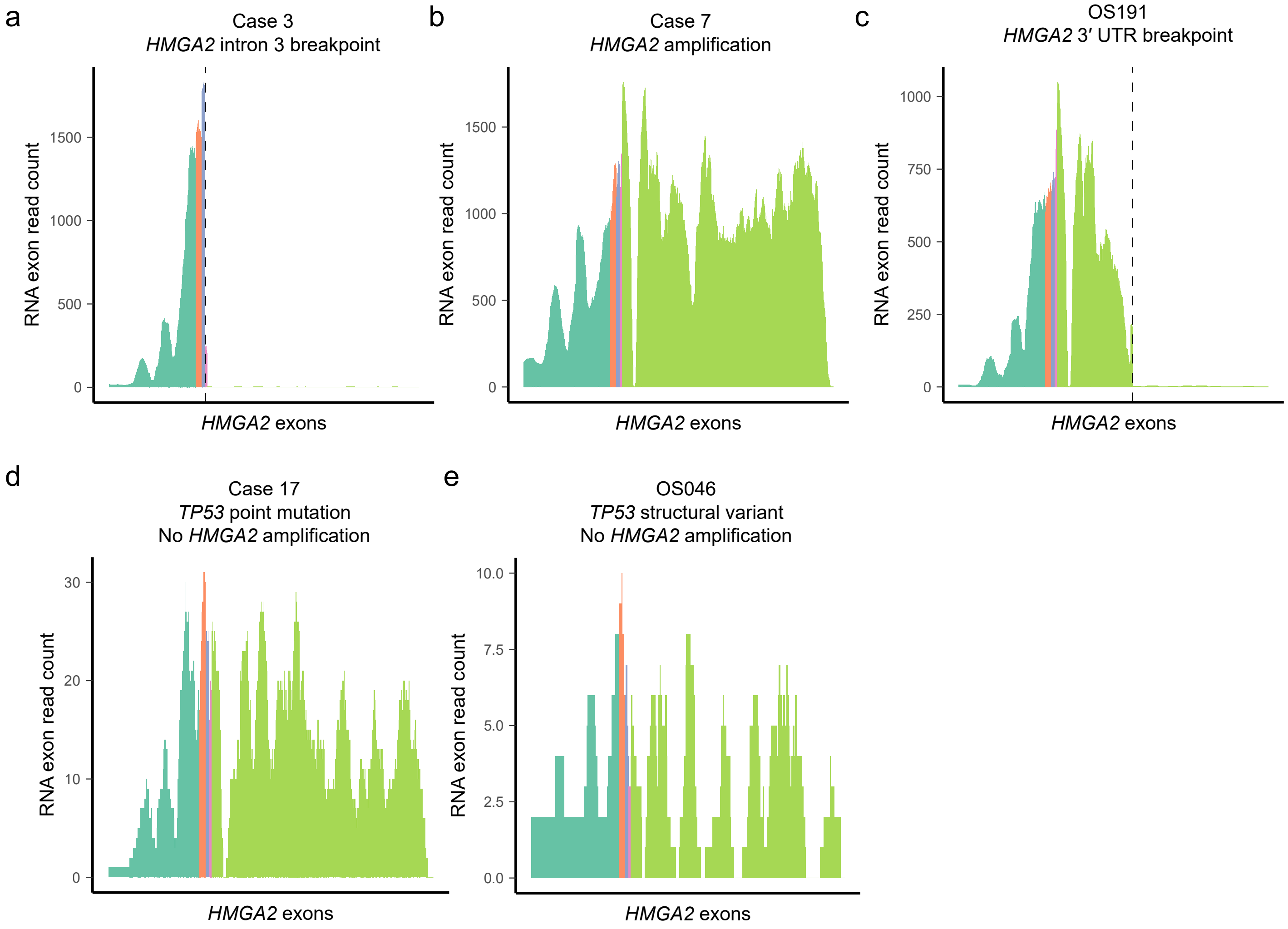

Supplementary Figure 23. *HMGA2* exon coverage plots in selected cases. Read coverage per exon of the *HMGA2* gene, with each exon depicted in a different colour. The dashed line indicates the breakpoint on the RNA level if applicable. *HMGA2* is either partially or fully amplified in *TP53*-wildtype cases (Supplementary Table S1) and examples are depicted in **a-c**. Cases with a partial amplification show a sharp decrease in exon coverage 3′ of detected breakpoint. *HMGA2* is not amplified in *TP53*-mutated cases, with examples shown in **d** and **e.**

Supplementary Figure 24

a b

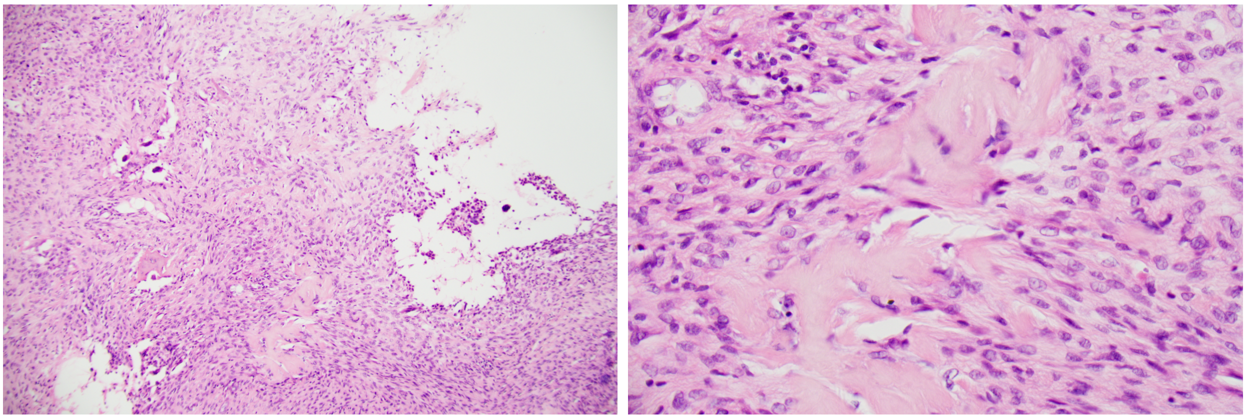

Supplementary Figure 24. Histological re-evaluation of Case 1. Photomicrographs of haematoxylin-eosin stained decalcified tumour tissue at x100 **(a)** and x400 **(b)** times magnification. The tumour consisted of moderately cellular fascicules of spindle cells with mild atypical features. Mitotic figures were rare. Throughout the tumour, neoplastic irregular bone could be identified **(b)**. The morphology was consistent with a low-grade central osteosarcoma.

Supplementary Table 1. Clinical and genetic features of *MDM2* and/or *CDK4* amplified osteosarcomas

Group	Case nr.	Age (years)	Sex	Diagnosis	Sample	Grade[7]	Location	Status and follow-up[8,9]	Selection criteria[10]
	Case 1	7	M	Low-grade central ostesarcoma[1]	Primary tumour	3	Femur	NED 167	Cohort 1: Gene expression
A	Case 2a	31	F	Parosteal osteosarcoma	Core needle biopsy	4	Femur	NED 23	Cohort 1: Amplification
	Case 2b				Primary tumour				
	Case 3	36	F	Parosteal osteosarcoma	Local recurrence	1	Femur	LR 42, NED 90	Cohort 1: Amplification
	Case 4	18	M	Parosteal osteosarcoma	Primary tumour	2	Femur	NED 215	Cohort 1: Diagnosis
	Case 5a	51	F	Low-grade central osteosarcoma	Open biopsy	NA	Femur	NED 44	Cohort 1: Amplification
	Case 5b				Primary tumour				
	Case 6	41	M	Parosteal osteosarcoma	Primary tumour	NA	Femur	NED 96	Cohort 1: Diagnosis
	Case 7	61	F	Parosteal osteosarcoma/Periosteal osteosarcoma, dedifferentiated[2]	Primary tumour	4	Femur	DoC 200	Cohort 1: Gene expression
	Case 8a	46	F	Parosteal osteosarcoma	Primary tumour	NA	Femur	M (soft tissue, multiple) 5, M (lymph node, multiple) 5, M (lung, multiple) 20, AwD 50	Cohort 1: Amplification
	Case 8b			Parosteal osteosarcoma, dedifferentiated?[3]	Metastasis		Subcutaneous		
B	Case 9a	43	F	Conventional osteosarcoma, fibroblastic	Primary tumour	NA	Sacrum	PD 22, DoD 34	Cohort 1: Amplification
	Case 9b				Persistent disease				
	Case 10	15	M	Parosteal osteosarcoma, dedifferentiated	Primary tumour	NA	Femur	NED 167	Cohort 1: Diagnosis
	OS191	17	F	Conventional osteosarcoma, osteoblastic[4]	Primary tumour	HG	Femur	NED 63	Cohort 2: Amplification
	Case 11	37	F	Low-grade central osteosarcoma[5]	Local recurrence	1-2	Mandible	NED 39	Cohort 1: Amplification
	Case 12a	30	F	Parosteal osteosarcoma, dedifferentiated	Primary tumour	NA	Fibula	M (lung) 36, LR 89, M (lung, multiple) 216, M (skeleton, multiple) 231, DoD 248	Cohort 1: Diagnosis
	Case 12b				Local recurrence				
	Case 12c				Metastasis		Lung		
	Case 13	26	M	Conventional osteosarcoma	Primary tumour	4	Pelvis	AwD 13	Cohort 1: Amplification
	Case 14	29	F	Conventional osteosarcoma, chondroblastic	Primary tumour	4	Digitorium 1, foot	NED 8	Cohort 1: Amplification
	OS181	9	F	Conventional osteosarcoma	Primary tumour	NA	Femur	NED 10	Cohort 2: Amplification
	Case 15	33	M	Conventional osteosarcoma, osteoblastic	Primary tumour	2-3	Femur	NED 126	Cohort 1: Gene expression
C	Case 16a	66	M	Conventional osteosarcoma[6]	Primary tumour	NA	Fibula	M (skeleton, multiple) 85, DoD 105	
	Case 16b				Metastasis		Skeleton		Cohort 1: Amplification
	OS131	15	M	Conventional osteosarcoma	Primary tumour	HG	Tibia	DoD	Cohort 2: Amplification
	Case 17a	10	M	Conventional osteosarcoma	Primary tumour	4	Femur	M (lung) 5, DoD 5	Cohort 1: Gene expression
	Case 17b				Metastasis		Lung		
	OS061	12	F	Conventional osteosarcoma	Primary tumour	HG	Femur	DoD	Cohort 2: Amplification
	OS188	8	F	Conventional osteosarcoma	Primary tumour	HG	Humerus	NED 25	Cohort 2: Amplification
D	OS117	8	F	Conventional osteosarcoma	Metastasis	HG	Femur	DoD	Cohort 2: Amplification
	OS222	17	M	Conventional osteosarcoma	Primary tumour	HG	Femur	NED 16	Cohort 2: Amplification
	OS046	10	M	Conventional osteosarcoma	Primary tumour	HG	Femur	DoD	Cohort 2: Amplification

Case nr.	SNP Array	Whole-genome mate pair sequencing	Single cell whole-genome sequencing	Longread whole-genome sequencing	RNA sequencing	Number of chromosomes affected by a copy number variant	Genomic Complexity Score[11]	Single Nucleotide Variants - selected	Structural variants - selected	Genomic copy number alterations - selected	*HMGA2* status	G-band karyotype	Case number in previous publication(s)
Case 1	Aberrant	Yes	No	Yes	Yes	1	0.29				Whole gene amplified	47-48,XY, +1-2r	PMID:16732325 Case 16 / PMID:38010733 Case 2
Case 2a	Aberrant	Yes	No	No	No	1	0.30				Whole gene amplified		
Case 2b	Aberrant	No	Yes	Yes	No	1	0.24				Whole gene amplified		
Case 3	Aberrant, noisy	No	No	No	Yes	1	0.35				5′ part amplified, breakpoint in intron 3		
Case 4	Aberrant	Yes	No	No	Yes	2	0.27				Whole gene amplified		
Case 5a	Aberrant	No	Yes	No	Yes	2	0.35		*FRS2::ANKRD62*		Whole gene amplified	47,XX,add(3)(q29),+12[9]/46,XX[2]	
Case 5b	Aberrant	Yes	No	No	Yes	2	0.27		*FRS2::ANKRD62*		Whole gene amplified		
Case 6	Aberrant	Yes	No	No	Yes	2	0.31		*FRS2::SINHCAF; FRS2::PPM1H*		5′ part amplified, breakpoint in intron 3	43-49,XY,+r,+1-2mar[cp1	PMID:16732325 Case 18
Case 7	Aberrant	Yes	No	Yes	Yes	18	0.38		*FRS2::ELF1*	*CDKN2A* homozygous deletion	Whole gene amplified	34-38,X,der(1)t(1;?;1)(q1	PMID:9790500 Case 1
Case 8a	Aberrant	Yes	Yes	Yes	Yes	16	0.33	*CDKN2A* c.C172T/p.R58X	*PLEKHA5::EPS8*	*CDKN2A* hemizygous deletion	Whole gene amplified	41,XX,add(1)(p?36),der(3;10)(q10;q10),add(5)(p15),add(5)(q35),add(7)(q?22),add(14)(p11),add(17)(p13),inc[cp2]/76-83,idemx2[cp2]/46,XX[3]	
Case 8b	Aberrant	No	No	No	No	15	0.31				Whole gene amplified		
Case 9a	Aberrant	No	No	No	Yes	4	0.38		*ALDH2::PLEKHA5::ATF7*	*TERT* gain	5′ part amplified, breakpoint in intron 3	39-45,XX,der(5)t(2;5)(q11;p15),-10,-15,add(15)(p11),-20,der(21;22)(q10;q10),+2mar[cp8]	
Case 9b	Aberrant	Yes	Yes	Yes	Yes	4	0.36		*ALDH2::PLEKHA5::ATF7*	*TERT* gain	5′ part amplified, breakpoint in intron 3		
Case 10	Aberrant	Yes	No	Yes	Yes	2	0.30		*FRS2::SSPN*		Whole gene amplified	48-50,XY,+1-5r[cp4]	
OS191	Aberrant	Yes	No	No	Yes	2	0.31		*FRS2* partners identified by RNAseq could not be verified on the DNA level		5′ part amplified, breakpoint in 3′ UTR		PMID:38010733 Case OS191
Case 11	Aberrant	No	No	No	Yes	7	0.29			*TERT* gain	5′ part amplified, breakpoint in 3′ UTR	46,XX[23]	
Case 12a	Aberrant, noisy	Yes	No	No	Yes	10	0.54		*FRS2::ADAM32*		Whole gene amplified	47, XX, +r/48,XX, +r, +ma	PMID:16732325 Case 14
Case 12b	Non-representative	No	No	No	No	Non-representative	NA				NA	46,XX	
Case 12c	Aberrant	No	No	Yes	No	11	0.38				Whole gene amplified	46,XX	
Case 13	Aberrant	Yes	Yes	Yes	No	23	0.50		*FRS2::DNAH2* (DNA)	*TERT* amplification	Whole gene amplified		
Case 14	Aberrant	No	No	Yes	No	23	0.48			*TERT* amplification	Whole gene amplified		
OS181	Aberrant	No	No	No	No	23	0.36				Whole gene amplified		PMID:38010733 Case OS181
Case 15	Aberrant	Yes	No	No	Yes	23	0.51				Whole gene amplified	45-50,X,-Y,del(1)(q21),ad	PMID:16732325 Case 17
Case 16a	Non-representative	No	No	No	No	Non-representative	NA				NA		
Case 16b	Aberrant	Yes	No	No	No	23	0.39				Whole gene amplified		
OS131	Aberrant	Yes	No	No	No	23	0.51		*FRS2::IRAK4* (DNA) and structural variant involving break in *TP53* intron 4		Hemizygous deletion		PMID:38010733 Case OS131
Case 17a	Aberrant	Yes	Yes	Yes	Yes	23	0.40	*TP53* c.C722T/p.S241F and c.G993T/p.Q331H	*FRS2* partners identified by RNAseq could not be verified on the DNA level		5′ part gained, breakpoint in intron 3		PMID:38010733 Case 5
Case 17b	High normal cell content	No	No	No	Yes	3	0.31	*TP53* c.C722T/p.S241F and c.G993T/p.Q331H	*FRS2* partners identified by RNAseq could not be verified on the DNA level		5′ part gained, breakpoint in intron 3		
OS061	Aberrant	Yes	No	No	Yes	23	0.41			*TP53* homozygous deletion	Hemizygous deletion		PMID:38010733 Case OS061
OS188	Aberrant	No	No	No	Yes	23	0.43		*TP53::NFYA (TP53* promoter gain)		Whole gene 1 copy gain		PMID:38010733 Case OS188
OS117	Aberrant	No	No	No	No	23	0.46		*TP53* promoter gain - unknown partner (SNP array)		5′ part gained, breakpoint in intron 3		PMID:38010733 Case OS117
OS222	Aberrant	Yes	No	No	Yes	21	0.44		*TP53::ELF1 (TP53* promoter gain)		Normal copy number		PMID:38010733 Case OS222
OS046	Aberrant	Yes	No	No	Yes	23	0.58		*TP53::E2F3 (TP53* promoter gain)		Normal copy number		PMID:38010733 Case OS046

[1]Case 1 was reviewed by F.HdF. The diagnosis was changed from conventional osteosarcoma to low-grade central osteosarcoma (Supplementary Figure 24).

[2]Dedifferentiated tumor diagnosed as periosteal osteosarcoma with histopathological features in line with parosteal osteosarcoma.

[3]Possible dedifferentiation.

[4]OS191 was reviewed by D.B. The diagnosis was not changed.

[5]Case 11 was initially diagnosed as a desmoplastic fibroma. The diagnosis was changed based on the post-operative biopsy obtained during surgical removal of the recurrence. Follow-up time is counted from the day the diagnosis was changed.

[6]Case 16 was diagnosed as a conventional osteosarcoma, with a note that it may be a dedifferentiated parosteal osteosarcoma. The diagnosis was not changed upon evaluation of the metastatic sample.

[7]Abbreviations: HG, high-grade; NA, not available.

[8]Abbreviations: DoD, dead of disease; NED, no evidence of disease; AwD, alive with disease; PD, persistent disease; DoC, dead of other causes; LR, local recurrence.

[9]Time in months after diagnosis.

[10]Cases were selected based on preexisting data from SNP array or RNA sequencing analyses. Cases with amplification and/or relative high gene expression levels of *CDK4* and/or *MDM2* were inlcuded, as well as cases diagnosed as parosteal or low-grade central osteosarcoma.

[11]Cases 3 and 12a had noisy SNP array profiles and Case 17b had a high normal cell content, resulting in artificially inflated and deflated genomic complexity scores, respectively. SNP array profiles from Cases 12b and 16a were deemed non-representative of tumour material and a genomic complexity score was not calculated.

Supplementary Table 2. Breakpoints affecting the *FRS2* and *PLEKHA5* genes identified by whole-genome mate pair and whole-genome longread sequencing

				**Breakpoints affecting *FRS2* and partner gene/region**					**Longread validation**		
Case nr.	**TIDDIT_SV_type[1]**	**Breakpoint_CHRA[2,3]**	**Breakpoint_partnerCHR[2,3]**	**Delly2_SV_type[1]**	**Breakpoint_CHRA[2,3]**	**Breakpoint_partnerCHR[2,3]**	**Manta_SV_type[1]**	**Breakpoint_CHRA[2,3]**	**Breakpoint_partnerCHR[2,3]**	**pbsv_SV_type[1]**	**Breakpoint_CHRA[3,4]**	**Breakpoint_partnerCHR[3,4]**
Case 5b	BND	**chr12:69867842**	**N[chr18:12041231[**	BND	**chr18:12041231**	**]chr12:69867843]T**	BND	**chr12:69866000**	**A[chr18:12043781[**	Longread sequencing not performed		
Case 6	INV	**chr12:31497783**	**chr12:69887681**	INV	**chr12:31494509**	**chr12:69887648**	Variant not detected			Longread sequencing not performed		
Case 7	BND	**chr12:69923907**	**N]chr13:41540297]**	BND	**chr13:41538976**	**T]chr12:69923906]**	Variant not detected			BND	**chr12:69530124**	**T]chr13:40966136]**
Case 9b	TDUP	chr12:69882800	chr12:92745007	DUP	chr12:69882797	chr12:92745009	TDUP	chr12:69882799	chr12:92745009	BND	chr12:69489020	]chr12:92351233]T
Case 10	INV	**chr12:26359568**	**chr12:72240079**	INV	**chr12:26359568**	**chr12:72240079**	Variant not detected			?[5]	**chr12:26205973**	**T[chr12:71846159[**
	INV	**chr12:69904397**	**chr12:72291420**	INV	**chr12:69904314**	**chr12:72290580**	Variant not detected			BND	**chr12:69510613**	**T]chr12:71897637]**
Case 12a	BND	**chr12:69926639**	**N[chr8:39062630[**	Variant not detected			BND	**chr12:69924779**	**T[chr8:39063426[**	Longread sequencing not performed		
Case12c	Mate pair sequencing not carried out									No structural variant in *FRS2* detected		
Case 13	BND	chr12:69873111	N]chr14:87746748]	BND	chr14:87746711	A]chr12:69873112]	BND	chr12:69873141	G]chr14:87746710]	BND	chr12:69479361	G]chr14:87280365]
	BND	**chr12:69948012**	**N[chr17:7656033[**	BND	**chr17:7656033**	**]chr12:69948013]A**	BND	**chr12:69946748**	**T[chr17:7656617[**	**BND**	**chr12:69554385**	**A[chr17:7752357[**
Case 16b	BND	chr12:69913120	[chr9:99406427[N	BND	chr12:69913120	[chr9:99406074[A	Variant not detected			Longread sequencing not performed		
OS131	DUP	**chr12:44130330**	**chr12:69920854**	DUP	**chr12:44130330**	**chr12:69920861**	TDUP	**chr12:44129986**	**chr12:69921403**	Longread sequencing not performed		
Case 17a	BND	chr12:69891384	N]chr6:57269113]	BND	chr12:69891392	G]chr6:57269114]	Variant not detected			BND	chr12:69497738	A]chr6:57404362]

				**Breakpoints affecting *PLEKHA5* and partner gene/region**					**Longread validation**		
Case nr.	**TIDDIT_SV_type[1]**	**Breakpoint_CHRA[2,3]**	**Breakpoint_partnerCHR[2,3]**	**Delly2_SV_type[1]**	**Breakpoint_CHRA[2,3]**	**Breakpoint_partnerCHR[2,3]**	**Manta_SV_type[1]**	**Breakpoint_CHRA[2,3]**	**Breakpoint_partnerCHR[2,3]**	**pbsv_SV_type[1]**	**Breakpoint_CHRA[3,4]**	**Breakpoint_partnerCHR[3,4]**
Case 8a	INV	**chr12:15885328**	**chr12:19375130**	INV	**chr12:15885184**	**chr12:19375120**	INV	**chr12:15885326**	**chr12:19375119**	BND	**chr12:15732392**	**T]chr12:19222183]**
Case 9b	TDUP	**chr12:19345498**	**chr12:112234234**	DUP	**chr12:19345498**	**chr12:112234205**	TDUP	**chr12:19349069**	**chr12:112231662**	BND	**chr12:19192480**	**]chr12:111796402]T**
	INV	**chr12:19472009**	**chr12:54002674**	INV	**chr12:19472005**	**chr12:53970592**	Variant not detected			BND	**chr12:19319071**	**A]chr12:53608858]**

[1]BND, break end; DUP, duplication; TDUP, tandem duplication; INV, inversion.

[2]All breakpoint positions are according to the GRCh37/hg19 build of the human reference genome.

[3]Structural variants highlighted in bold are those where either the *FRS2* or *PLEKHA5* regulatory regions can come to control another gene or partner region.

[4]All breakpoint positions are according to the GRCh38/hg38 build of the human reference genome.

[5]The variant is not detected by pbsv as it only had two supporting reads upon manual inspection of the BAM file.

Supplementary Table 3. *FRS2* and *PLEKHA5* fusion transcripts detected by FusionCatcher and STAR-Fusion

Algorithm	FusionCatcher															
Case nr	Gene_1_symbol(5end_fusion_partner)	Gene_2_symbol(3end_fusion_partner)	Fusion_description	Counts_of	Spanning_	Spanning_	Longest_a	Fusion_finding_method	Fusion_point_for_gene_1(5end_fusion_partner)[1]	Fusion_point_for_gene_2(3end_fusion_partner)[1]	Gene_1_id(5end_fusion_partner)	Gene_2_id(3end_fusion_partner)	Exon_1_id(5end_fusion_partner)	Exon_2_id(3end_fusion_partner)	Fusion_sequence	Predicted_effect
Case 5a[2]	FRS2	AP002414.5	lincrna,exon-exon	0	73	16	27	BOWTIE;BOWTIE+BLAT;BOWTIE+STAR	12:69864310:+	18:12081796:+	ENSG00000166225	ENSG00000267722	ENSE00001206507	ENSE00002737324	CCGCCGACCCGCGCCCTGCTCCCTCTCAGCACCTGGGCGGACG*GCTCAACATGGGAAAAGACATTCCGGCAGATCGGCTTTGAAAG	UTR/exonic(no-known-CDS)
Case 5b[2]	FRS2	AP002414.5	lincrna,exon-exon	0	16	5	26	BOWTIE	12:69864310:+	18:12081796:+	ENSG00000166225	ENSG00000267722	ENSE00001206507	ENSE00002737324	CCGCCGACCCGCGCCCTGCTCCCTCTCAGCACCTGGGCGGACG*GCTCAACATGGGAAAAGACATTCCGGCAGATCGGCTTTGAAAG	UTR/exonic(no-known-CDS)
Case 6[3]	FRS2	FAM60A	m447,exon-exon	0	1216	29	29	BOWTIE;BOWTIE+BLAT;BOWTIE+STAR	12:69864310:+	12:31451158:-	ENSG00000166225	ENSG00000139146	ENSE00001206507	ENSE00003661117	TTCGCGCCCGCCGACCCGCGCCCTGCTCCCTCTCAGCACCTGGGCGGACG*ACTATTGCCCAGAAGAAAAGATGTTTGGTTTTCACAAGCCAAAGATGTAC	UTR/UTR
	FRS2	FAM60A	m447,exon-exon	0	1216	25	30	BOWTIE;BOWTIE+BLAT;BOWTIE+STAR	12:69864310:+	12:31458057:-	ENSG00000166225	ENSG00000139146	ENSE00001206507	ENSE00002265807	CCGCCGACCCGCGCCCTGCTCCCTCTCAGCACCTGGGCGGACG*GCACAACTTCCTGAATGGACCATGACTCCCACCAAAGATCCCT	UTR/UTR
	FRS2	FAM60A	m447,exon-exon	0	1216	21	30	BOWTIE;BOWTIE+BLAT;BOWTIE+STAR	12:69880077:+	12:31451158:-	ENSG00000166225	ENSG00000139146	ENSE00002340788	ENSE00003661117	CCAGCCAGCGTGTGCTTACTTCATGTCTCTGGGTCACATTTTG*ACTATTGCCCAGAAGAAAAGATGTTTGGTTTTCACAAGCCAAA	UTR/UTR
	FRS2	FAM60A	m447,exon-exon	0	1216	8	29	BOWTIE;BOWTIE+STAR	12:69885431:+	12:31451158:-	ENSG00000166225	ENSG00000139146	ENSE00002399617	ENSE00003661117	TTCTGACTCAGCCTCCCAAGTAGGTAGGACCACAGGTGTACAG*ACTATTGCCCAGAAGAAAAGATGTTTGGTTTTCACAAGCCAAA	UTR/UTR
	FRS2	FAM60A	m447,exon-exon	0	1216	6	30	BOWTIE	12:69880077:+	12:31458057:-	ENSG00000166225	ENSG00000139146	ENSE00002340788	ENSE00002265807	CCAGCCAGCGTGTGCTTACTTCATGTCTCTGGGTCACATTTTG*GCACAACTTCCTGAATGGACCATGACTCCCACCAAAGATCCCT	UTR/UTR
	FRS2	PPM1H	exon-exon	0	84	23	29	BOWTIE;BOWTIE+BLAT;BOWTIE+STAR	12:69864310:+	12:63087779:-	ENSG00000166225	ENSG00000111110	ENSE00001206507	ENSE00003631877	CCGCCGACCCGCGCCCTGCTCCCTCTCAGCACCTGGGCGGACG*GGCATACAAAACCATTGAGGATGAGGACTTGAAGTTCCCCCTT	UTR/CDS(truncated)
Case 7	FRS2	ELF1	exon-exon	0	2104	27	30	BOWTIE;BOWTIE+BLAT;BOWTIE+STAR	12:69864310:+	13:41533152:-	ENSG00000166225	ENSG00000120690	ENSE00001206507	ENSE00003481363	CCGCCGACCCGCGCCCTGCTCCCTCTCAGCACCTGGGCGGACG*CTTGGTGATCCAGCTATTTTTCCTGCCGTAATTGTGGAACATG	UTR/CDS(truncated)
	FRS2	ELF1	exon-exon	0	2104	15	30	BOWTIE;BOWTIE+BLAT;BOWTIE+STAR	12:69874139:+	13:41533152:-	ENSG00000166225	ENSG00000120690	ENSE00002397718	ENSE00003481363	TGAGTTTCTGCCTCTACAAATTTGACTATTCTAGGTACTTCAT*CTTGGTGATCCAGCTATTTTTCCTGCCGTAATTGTGGAACATG	UTR/CDS(truncated)
	FRS2	ELF1	exon-exon	0	2104	13	29	BOWTIE;BOWTIE+STAR	12:69885431:+	13:41533152:-	ENSG00000166225	ENSG00000120690	ENSE00002399617	ENSE00003481363	TTCTGACTCAGCCTCCCAAGTAGGTAGGACCACAGGTGTACAG*CTTGGTGATCCAGCTATTTTTCCTGCCGTAATTGTGGAACATG	UTR/CDS(truncated)
	FRS2	ELF1		0	2104	8	33	BOWTIE+BLAT;BOWTIE+STAR	12:69867988:+	13:41533152:-	ENSG00000166225	ENSG00000120690			GTGTCGGAGATGTTGACGGTGGTACTCTTACTAGAAATACGGGCAAGGAG*CTTGGTGATCCAGCTATTTTTCCTGCCGTAATTGTGGAACATGTTCCTGG	intronic/CDS(truncated)
	FRS2	ELF1	exon-exon	0	2104	8	25	BOWTIE+BLAT;BOWTIE+STAR	12:69880077:+	13:41533152:-	ENSG00000166225	ENSG00000120690			CATTTTTCCAGCCAGCGTGTGCTTACTTCATGTCTCTGGGTCACATTTTG*CTTGGTGATCCAGCTATTTTTCCTGCCGTAATTGTGGAACATGTTCCTGG	UTR/CDS(truncated)
Case 8a	PLEKHA5	EPS8	oncogene,tcga,chimerdb3seq,cancer,tcga-cancer,exon-exon	0	117	22	29	BOWTIE;BOWTIE+BLAT;BOWTIE+STAR	12:19358377:+	12:15835906:-	ENSG00000052126	ENSG00000151491	ENSE00001742305	ENSE00001284194	GAGACAAAGAAAAGAACACAGATTTTTAGCAAAAGGCTATTTG*AACACACAAGTGAAAGACACAATGAATGGTCATATTTCTAATC	UTR/UTR
	PLEKHA5	EPS8	oncogene,tcga,chimerdb3seq,cancer,tcga-cancer	0	117	5	38	BOWTIE+BLAT;BOWTIE+STAR	12:19358377:+	12:15866259:-	ENSG00000052126	ENSG00000151491			GAAGCCTGAGACAAAGAAAAGAACACAGATTTTTAGCAAAAGGCTATTTG*TGTCTGTAGTGGGAAGTCTTCATGAGCTCAGTTGGCCATATTTCTGTACC	UTR/intronic
Case 9a	PLEKHA5	ATF7	exon-exon	0	1101	27	30	BOWTIE	12:19440490:+	12:53994805:-	ENSG00000052126	ENSG00000170653	ENSE00003464053	ENSE00003471393	GACTTTACAGTCTGTTAGTCCCCAGAGCCTCCAAGGGAAAACG*CTCCTCTCTGCTATATGAAATATGGGAGACGACAGACCGTTTG	CDS(truncated)/UTR
	PLEKHA5	ATF7	exon-exon	0	1101	21	30	BOWTIE	12:19440490:+	12:53946421:-	ENSG00000052126	ENSG00000170653	ENSE00003464053	ENSE00003624511	GACTTTACAGTCTGTTAGTCCCCAGAGCCTCCAAGGGAAAACG*AGATTTACAAACGAGGACCACCTGGCAGTTCATAAACACAAGC	in-frame
	PLEKHA5	ATF7	exon-exon	0	1101	20	29	BOWTIE	12:19436679:+	12:53946421:-	ENSG00000052126	ENSG00000170653	ENSE00003465943	ENSE00003624511	AGATGTGACAATAGACCGCAGACACAGGGCCCATCACCCTAAG*AGATTTACAAACGAGGACCACCTGGCAGTTCATAAACACAAGC	in-frame
	PLEKHA5	ATF7	exon-exon	0	1101	18	29	BOWTIE	12:19436679:+	12:53994805:-	ENSG00000052126	ENSG00000170653	ENSE00003465943	ENSE00003471393	AGATGTGACAATAGACCGCAGACACAGGGCCCATCACCCTAAG*CTCCTCTCTGCTATATGAAATATGGGAGACGACAGACCGTTTG	CDS(truncated)/UTR
	PLEKHA5	ATF7	exon-exon	0	1101	6	29	BOWTIE	12:19467828:+	12:53994805:-	ENSG00000052126	ENSG00000170653	ENSE00001775395	ENSE00003471393	AATGATTGAGAACTCGGCGCTAAGACCCCAACTGTACCAGCAA*CTCCTCTCTGCTATATGAAATATGGGAGACGACAGACCGTTTG	CDS(truncated)/UTR
	PLEKHA5	ATF7	exon-exon	0	1101	5	29	BOWTIE	12:19444631:+	12:53994805:-	ENSG00000052126	ENSG00000170653	ENSE00001295918	ENSE00003471393	TTCACATCATCTCCAAAGGAACACCATATATTTGGATCATCAG*CTCCTCTCTGCTATATGAAATATGGGAGACGACAGACCGTTTG	CDS(truncated)/UTR
	ALDH2	PLEKHA5		12	215	21	37	BOWTIE;BOWTIE+BLAT;BOWTIE+STAR	12:112230586:+	12:19353259:+	ENSG00000111275	ENSG00000052126	ENSE00003667483	ENSE00002312709	CTCGGGTGGTCGGGAACCCCTTTGATAGCAAGACCGAGCAGGGGCCGCAG*CTGAATAGATTTCCTTTTCATTCTTCCTGGGAATAGAAGATGTAATGGAA	out-of-frame
	ALDH2	PLEKHA5		12	215	19	37	BOWTIE+BLAT	12:112230584:+	12:19353259:+	ENSG00000111275	ENSG00000052126			CTCGGGTGGTCGGGAACCCCTTTGATAGCAAGACCGAGCAGGGGCCGC*CTGAATAGATTTCCTTTTCATTCTTCCTGGGAATAGAAGATGTAATGGAA	out-of-frame
	ALDH2	PLEKHA5		12	215	10	38	BOWTIE;BOWTIE+BLAT;BOWTIE+STAR	12:112230586:+	12:19360200:+	ENSG00000111275	ENSG00000052126	ENSE00003667483	ENSE00002234415	CTCGGGTGGTCGGGAACCCCTTTGATAGCAAGACCGAGCAGGGGCCGCAG*TCCCAGATACCTCTAATCATAAGTCTTCATAGATCAATAGAAGAGAAATA	CDS(truncated)/UTR
	ALDH2	PLEKHA5		12	215	3	26	BOWTIE	12:112230586:+	12:19406874:+	ENSG00000111275	ENSG00000052126	ENSE00003667483	ENSE00003604713	GGTCGGGAACCCCTTTGATAGCAAGACCGAGCAGGGGCCGCAG*CCATAATGAAAGGAAAGTGACCTGCAAACATCCAGTCACAGGA	out-of-frame
Case 9b	PLEKHA5	ATF7	exon-exon	0	2457	27	30	BOWTIE	12:19440490:+	12:53994805:-	ENSG00000052126	ENSG00000170653	ENSE00003464053	ENSE00003471393	GACTTTACAGTCTGTTAGTCCCCAGAGCCTCCAAGGGAAAACG*CTCCTCTCTGCTATATGAAATATGGGAGACGACAGACCGTTTG	CDS(truncated)/UTR
	PLEKHA5	ATF7	exon-exon	0	2457	26	30	BOWTIE	12:19436679:+	12:53946421:-	ENSG00000052126	ENSG00000170653	ENSE00003465943	ENSE00003624511	AGATGTGACAATAGACCGCAGACACAGGGCCCATCACCCTAAG*AGATTTACAAACGAGGACCACCTGGCAGTTCATAAACACAAGC	in-frame
	PLEKHA5	ATF7	exon-exon	0	2457	25	30	BOWTIE;BOWTIE+STAR	12:19440490:+	12:53946421:-	ENSG00000052126	ENSG00000170653	ENSE00003464053	ENSE00003624511	GACTTTACAGTCTGTTAGTCCCCAGAGCCTCCAAGGGAAAACG*AGATTTACAAACGAGGACCACCTGGCAGTTCATAAACACAAGC	in-frame
	PLEKHA5	ATF7	exon-exon	0	2457	23	30	BOWTIE;BOWTIE+STAR	12:19436679:+	12:53994805:-	ENSG00000052126	ENSG00000170653	ENSE00003465943	ENSE00003471393	AGATGTGACAATAGACCGCAGACACAGGGCCCATCACCCTAAG*CTCCTCTCTGCTATATGAAATATGGGAGACGACAGACCGTTTG	CDS(truncated)/UTR
	PLEKHA5	ATF7	exon-exon	0	2457	11	29	BOWTIE	12:19467828:+	12:53994805:-	ENSG00000052126	ENSG00000170653	ENSE00001775395	ENSE00003471393	AATGATTGAGAACTCGGCGCTAAGACCCCAACTGTACCAGCAA*CTCCTCTCTGCTATATGAAATATGGGAGACGACAGACCGTTTG	CDS(truncated)/UTR
	PLEKHA5	ATF7	exon-exon	0	2457	5	29	BOWTIE	12:19444631:+	12:53994805:-	ENSG00000052126	ENSG00000170653	ENSE00001295918	ENSE00003471393	TTCACATCATCTCCAAAGGAACACCATATATTTGGATCATCAG*CTCCTCTCTGCTATATGAAATATGGGAGACGACAGACCGTTTG	CDS(truncated)/UTR
	ALDH2	PLEKHA5	cancer,exon-exon	10	798	27	30	BOWTIE;BOWTIE+BLAT;BOWTIE+STAR	12:112230586:+	12:19353259:+	ENSG00000111275	ENSG00000052126	ENSE00003667483	ENSE00002312709	GGTCGGGAACCCCTTTGATAGCAAGACCGAGCAGGGGCCGCAG*CTGAATAGATTTCCTTTTCATTCTTCCTGGGAATAGAAGATGT	out-of-frame
	ALDH2	PLEKHA5	cancer,exon-exon	10	798	26	30	BOWTIE;BOWTIE+BLAT;BOWTIE+STAR	12:112230586:+	12:19360200:+	ENSG00000111275	ENSG00000052126	ENSE00003667483	ENSE00002234415	GGTCGGGAACCCCTTTGATAGCAAGACCGAGCAGGGGCCGCAG*TCCCAGATACCTCTAATCATAAGTCTTCATAGATCAATAGAAG	CDS(truncated)/UTR
	ALDH2	PLEKHA5	cancer,exon-exon	10	798	6	26	BOWTIE	12:112230586:+	12:19406874:+	ENSG00000111275	ENSG00000052126	ENSE00003667483	ENSE00003604713	GGTCGGGAACCCCTTTGATAGCAAGACCGAGCAGGGGCCGCAG*CCATAATGAAAGGAAAGTGACCTGCAAACATCCAGTCACAGGA	out-of-frame
Case 10	FRS2	SSPN	cancer,exon-exon	0	59	9	27	BOWTIE;BOWTIE+BLAT;BOWTIE+STAR	12:69864310:+	12:26377226:+	ENSG00000166225	ENSG00000123096	ENSE00001206507	ENSE00003664807	CCGCCGACCCGCGCCCTGCTCCCTCTCAGCACCTGGGCGGACG*GTCTGCTTAGTGGCCTATCTTGGCTTGTTTATGCTTTGTGTCT	UTR/CDS(truncated)
OS191[4]	FRS2	CNTN1	exon-exon	0	33	14	30	BOWTIE;BOWTIE+BLAT;BOWTIE+STAR	12:69864310:+	12:41302159:+	ENSG00000166225	ENSG00000018236	ENSE00001206507	ENSE00002387517	CCGCCGACCCGCGCCCTGCTCCCTCTCAGCACCTGGGCGGACG*GTGTTTAAAATTATCCAACTGCCATAGAGCTAAATTCTTTTTT	UTR/UTR
	FRS2	CNTN1	exon-exon	0	33	3	27	BOWTIE	12:69864310:+	12:41289752:+	ENSG00000166225	ENSG00000018236	ENSE00001206507	ENSE00002416175	CCGCCGACCCGCGCCCTGCTCCCTCTCAGCACCTGGGCGGACG*TAGAGACGGGGTTTCACCGTGTTAGCCAAGATGGTCTCGATCT	UTR/UTR
	FRS2	GSTA2	m7,exon-exon	0	10	4	28	BOWTIE	12:69864310:+	6:52622775:-	ENSG00000166225	ENSG00000244067	ENSE00001206507	ENSE00002460854	CCGCCGACCCGCGCCCTGCTCCCTCTCAGCACCTGGGCGGACG*GCTTAGAGAAACCTCCAGGAGACTGCTACCATGGCAGAGAAGC	UTR/UTR
	FRS2	GRIP1	tcga,chimerdb3seq,tcga-cancer	0	7	4	20	BOWTIE+STAR	12:69864310:+	12:66776001:-	ENSG00000166225	ENSG00000155974			TTCGCGCCCGCCGACCCGCGCCCTGCTCCCTCTCAGCACCTGGGCGGACG*GATCTCACTTTGTCACCCAGACTGGAGTGCAGTGGTGCAAACTTGGCTCA	UTR/intronic
	FRS2	GRIP1	tcga,chimerdb3seq,tcga-cancer,exon-exon	0	7	3	26	BOWTIE	12:69864310:+	12:66990707:-	ENSG00000166225	ENSG00000155974	ENSE00001206507	ENSE00003671421	CCGCCGACCCGCGCCCTGCTCCCTCTCAGCACCTGGGCGGACG*ATGAGAGTCCCTACACTAAATCCGCCAGCCAGACAAAGCCGCC	UTR/CDS(truncated)
Case 12a	FRS2	ADAM32	exon-exon	0	119	18	29	BOWTIE	12:69925836:+	8:39068663:+	ENSG00000166225	ENSG00000197140	ENSE00001724775	ENSE00001800049	GAACAAAATTGTATCTGTTTTTCTCAGAAGAGAATTCCACAAG*GCAATCCAATGGTGTGAAGACTTTTAGCAGTTGCAGTTTGAGG	UTR/CDS(truncated)
	FRS2	ADAM32	exon-exon	0	119	14	30	BOWTIE;BOWTIE+BLAT;BOWTIE+STAR	12:69925836:+	8:39079129:+	ENSG00000166225	ENSG00000197140	ENSE00001724775	ENSE00003651540	GAACAAAATTGTATCTGTTTTTCTCAGAAGAGAATTCCACAAG*CAATGTGGACCTGCAAGCTGTTGTGATTTTCGAACTTGTGTAC	UTR/CDS(truncated)
Case 17a[4]	FRS2	AL136311.1	antisense,exon-exon	0	40	16	30	BOWTIE;BOWTIE+BLAT;BOWTIE+STAR	12:69864310:+	6:57035896:-	ENSG00000166225	ENSG00000226803	ENSE00001206507	ENSE00001659420	CCGCCGACCCGCGCCCTGCTCCCTCTCAGCACCTGGGCGGACG*ATCCACACCATTAGTAAACCCCATCAATTTTGCCTTCAGAATA	UTR/exonic(no-known-CDS)
Case 17b[4]	FRS2	AL136311.1	antisense,exon-exon	0	10	5	22	BOWTIE;BOWTIE+BLAT;BOWTIE+STAR	12:69880077:+	6:57035896:-	ENSG00000166225	ENSG00000226803	ENSE00002340788	ENSE00001659420	CCAGCCAGCGTGTGCTTACTTCATGTCTCTGGGTCACATTTTG*ATCCACACCATTAGTAAACCCCATCAATTTTGCCTTCAGAATA	UTR/exonic(no-known-CDS)

[1]All breakpoint positions are according to the GRCh37/hg19 build of the human reference genome.

[2]The nearest coding gene in the same sense is *ANKRD62*.

[3]The *FAM60A* gene has been renamed to *SINHCAF*.

[4]The 3′ partners of *FRS2* in OS191 and Case 17 could not be verified on the DNA level.

Supplementary Table 3. *FRS2* and *PLEKHA5* fusion transcripts detected by FusionCatcher and STAR-Fusion (continued)

Algorithm	STAR-Fusion													
Case nr	#FusionName	JunctionReadCount	SpanningFragCount	SpliceType	LeftGene	LeftBreakpoint[1]	RightGene	RightBreakpoint[1]	LargeAnchorSupport	LeftBreakDinuc	LeftBreakEntropy	RightBreakDinuc	RightBreakEntropy	annots
Case 5a[2]	FRS2--RP11-815J4.1	7	0	ONLY_REF_SPLICE	FRS2^ENSG00000166225.4	chr12:69864310:+	RP11-815J4.1^ENSG00000267722.1	chr18:12081796:+	YES_LDAS	GT	1.6895	AG	1.8892	["INTERCHROMOSOMAL[chr12--chr18]"]
Case 5b[2]	Fusion not identified													
Case 6[3]	FRS2--FAM60A	35	19	ONLY_REF_SPLICE	FRS2^ENSG00000166225.4	chr12:69864310:+	FAM60A^ENSG00000139146.9	chr12:31451158:-	YES_LDAS	GT	1.6895	AG	1.9656	["INTRACHROMOSOMAL[chr12:38.38Mb]"]
	FRS2--FAM60A	20	19	ONLY_REF_SPLICE	FRS2^ENSG00000166225.4	chr12:69864310:+	FAM60A^ENSG00000139146.9	chr12:31458057:-	YES_LDAS	GT	1.6895	AG	1.9086	["INTRACHROMOSOMAL[chr12:38.38Mb]"]
	FRS2--FAM60A	6	19	ONLY_REF_SPLICE	FRS2^ENSG00000166225.4	chr12:69885431:+	FAM60A^ENSG00000139146.9	chr12:31451158:-	YES_LDAS	GT	1.9329	AG	1.9656	["INTRACHROMOSOMAL[chr12:38.38Mb]"]
	FRS2--FAM60A	6	19	ONLY_REF_SPLICE	FRS2^ENSG00000166225.4	chr12:69880077:+	FAM60A^ENSG00000139146.9	chr12:31451158:-	YES_LDAS	GT	1.8892	AG	1.9656	["INTRACHROMOSOMAL[chr12:38.38Mb]"]
	FRS2--FAM60A	4	19	ONLY_REF_SPLICE	FRS2^ENSG00000166225.4	chr12:69880077:+	FAM60A^ENSG00000139146.9	chr12:31458057:-	YES_LDAS	GT	1.8892	AG	1.9086	["INTRACHROMOSOMAL[chr12:38.38Mb]"]
	FRS2--PPM1H	6	0	ONLY_REF_SPLICE	FRS2^ENSG00000166225.4	chr12:69864310:+	PPM1H^ENSG00000111110.7	chr12:63087779:-	YES_LDAS	GT	1.6895	AG	1.7968	["INTRACHROMOSOMAL[chr12:6.54Mb]"]
Case 7	FRS2--ELF1	261	53	ONLY_REF_SPLICE	FRS2^ENSG00000166225.4	chr12:69864310:+	ELF1^ENSG00000120690.9	chr13:41533152:-	YES_LDAS	GT	1.6895	AG	1.9329	["INTERCHROMOSOMAL[chr12--chr13]"]
	FRS2--ELF1	95	53	ONLY_REF_SPLICE	FRS2^ENSG00000166225.4	chr12:69880077:+	ELF1^ENSG00000120690.9	chr13:41533152:-	YES_LDAS	GT	1.8892	AG	1.9329	["INTERCHROMOSOMAL[chr12--chr13]"]
Case 8a	PLEKHA5--EPS8	15	6	ONLY_REF_SPLICE	PLEKHA5^ENSG00000052126.10	chr12:19358377:+	EPS8^ENSG00000151491.8	chr12:15835906:-	YES_LDAS	GT	1.9329	AG	1.5546	["INTRACHROMOSOMAL[chr12:3.25Mb]"]
Case 9a	Fusion not identified													
	ALDH2--PLEKHA5	68	16	ONLY_REF_SPLICE	ALDH2^ENSG00000111275.8	chr12:112230586:+	PLEKHA5^ENSG00000052126.10	chr12:19353259:+	YES_LDAS	GT	1.4566	AG	1.8892	["INTRACHROMOSOMAL[chr12:92.68Mb]"]
	ALDH2--PLEKHA5	26	16	ONLY_REF_SPLICE	ALDH2^ENSG00000111275.8	chr12:112230586:+	PLEKHA5^ENSG00000052126.10	chr12:19360200:+	YES_LDAS	GT	1.4566	AG	1.8062	["INTRACHROMOSOMAL[chr12:92.68Mb]"]
Case 9b	Fusion not identified													
	ALDH2--PLEKHA5	100	67	ONLY_REF_SPLICE	ALDH2^ENSG00000111275.8	chr12:112230586:+	PLEKHA5^ENSG00000052126.10	chr12:19353259:+	YES_LDAS	GT	1.4566	AG	1.8892	["INTRACHROMOSOMAL[chr12:92.68Mb]"]
Case 10	FRS2--SSPN	4	1	ONLY_REF_SPLICE	FRS2^ENSG00000166225.4	chr12:69864310:+	SSPN^ENSG00000123096.7	chr12:26377226:+	YES_LDAS	GT	1.6895	AG	1.8256	["INTRACHROMOSOMAL[chr12:43.41Mb]"]
OS191[4]	FRS2--CNTN1	3	2	ONLY_REF_SPLICE	FRS2^ENSG00000166225.4	chr12:69864310:+	CNTN1^ENSG00000018236.10	chr12:41302159:+	YES_LDAS	GT	1.6895	AG	1.6895	["INTRACHROMOSOMAL[chr12:28.40Mb]"]
	FRS2--GSTA2	2	0	ONLY_REF_SPLICE	FRS2^ENSG00000166225.4	chr12:69864310:+	GSTA2^ENSG00000244067.1	chr6:52622775:-	YES_LDAS	GT	1.6895	AG	1.9656	["INTERCHROMOSOMAL[chr12--chr6]"]
	Fusion not identified													
Case 12a	FRS2--ADAM32	13	8	ONLY_REF_SPLICE	FRS2^ENSG00000166225.4	chr12:69925836:+	ADAM32^ENSG00000197140.10	chr8:39079129:+	YES_LDAS	GT	1.8295	AG	1.9899	["INTERCHROMOSOMAL[chr12--chr8]"]
	FRS2--ADAM32	12	8	ONLY_REF_SPLICE	FRS2^ENSG00000166225.4	chr12:69925836:+	ADAM32^ENSG00000197140.10	chr8:39068663:+	YES_LDAS	GT	1.8295	AG	1.9899	["INTERCHROMOSOMAL[chr12--chr8]"]
Case 17a[4]	FRS2--RP11-203B9.4	1	1	ONLY_REF_SPLICE	FRS2^ENSG00000166225.4	chr12:69864310:+	RP11-203B9.4^ENSG00000226803.3	chr6:57035896:-	YES_LDAS	GT	1.6895	AG	1.8256	["INTERCHROMOSOMAL[chr12--chr6]"]
Case 17b[4]	Fusion not identified													

[1]All breakpoint positions are according to the GRCh37/hg19 build of the human reference genome.

[2]The nearest coding gene in the same sense is *ANKRD62* .

[3]The *FAM60A* gene has been renamed to *SINHCAF* .

[4]The 3′ partners of *FRS2* in OS191 and Case 17 could not be verified on the DNA level.
